# Identifying Resilience Factors of Distress and Paranoia During the COVID-19 Outbreak in Five Countries

**DOI:** 10.3389/fpsyg.2021.661149

**Published:** 2021-06-10

**Authors:** Martin Jensen Mækelæ, Niv Reggev, Renata P. Defelipe, Natalia Dutra, Ricardo M. Tamayo, Kristoffer Klevjer, Gerit Pfuhl

**Affiliations:** ^1^Department of Psychology, UiT the Arctic University of Norway, Tromsø, Norway; ^2^Department of Psychology, Zlotowski Center for Neuroscience, Ben Gurion University of the Negev, Beersheba, Israel; ^3^Instituto de Psicologia, Universidade de São Paulo, São Paulo, Brazil; ^4^Evolution of Human Behavior Laboratory, Department of Physiology and Behavior, Universidade Federal do Rio Grande do Norte, Natal, Brazil; ^5^Departamento de Psicología, Universidad Nacional de Colombia, Bogotá, Colombia

**Keywords:** pandemic (COVID-19), coping behavior, thriving, protective factor, mental health

## Abstract

The ongoing COVID-19 pandemic outbreak has affected all countries with more than 100 million confirmed cases and over 2.1 million casualties by the end of January 2021 worldwide. A prolonged pandemic can harm global levels of optimism, regularity, and sense of meaning and belonging, yielding adverse effects on individuals' mental health as represented by worry, paranoia, and distress. Here we studied resilience, a successful adaptation despite risk and adversity, in five countries: Brazil, Colombia, Germany, Israel, and Norway. In April 2020, over 2,500 participants were recruited for an observational study measuring protective and obstructive factors for distress and paranoia. More than 800 of these participants also completed a follow-up study in July. We found that thriving, keeping a regular schedule, engaging in physical exercise and less procrastination served as factors protecting against distress and paranoia. Risk factors were financial worries and a negative mindset, e.g., feeling a lack of control. Longitudinally, we found no increase in distress or paranoia despite an increase in expectation of how long the outbreak and the restrictions will last, suggesting respondents engaged in healthy coping and adapting their lives to the new circumstances. Altogether, our data suggest that humans adapt even to prolonged stressful events. Our data further highlight several protective factors that policymakers should leverage when considering stress-reducing policies.

## Introduction

On 31 December 2019, China informed the World Health Organization (WHO) about cases of pneumonia with unknown etiology, later connected to the SARS-CoV-2 virus. The Coronavirus spread globally and on the 11th of March 2020 the WHO classified it as a global pandemic. Even though governments around the world employed various countermeasures in an attempt to contain the virus, the pandemic evolved into a severe global health problem (Adhanom Ghebreyesus, 2020), threatening to lead to a temporary collapse of numerous local healthcare systems (Zhu and Peng, 2019; WHO, 2020).

Designed as protective measures for physical health, these countermeasures drastically changed the lifestyle of most members of present-day societies by recommending and even enforcing social isolation. Such abrupt changes in everyday life inevitably led to a heightened sense of personal and societal uncertainty, increasing mental illness and distress worldwide (Mækelæ et al., 2020; Okruszek et al., 2020; Torales et al., 2020; Xiang et al., 2020). Indeed, a longitudinal study comparing the distress of US citizens before and at an early stage of the pandemic found three times more depression and anxiety during the pandemic (Twenge and Joiner, 2020). Mental health is a multifaceted construct defined as more than the mere absence of illness (Foundation, 2005). Mental health relies on two distinct yet correlated dimensions of mental illness and positive mental health (Westerhof and Keyes, 2010; Provencher and Keyes, 2011). Elevated levels of illness can coincide with high levels of well-being, but the absence of illness does not imply the presence of well-being, and vice versa (Sin and Lyubomirsky, 2009). Mental illness can manifest as affective, anxiety and personality disorders (e.g., depression, anxiety, paranoia) or feelings of distress. These disorders, in turn, are linked to negative health outcomes as well as impaired mental, physical and social functioning (Corey, 2002; Sun et al., 2019). Positive mental health is multifaceted and encompasses both hedonic- and eudaimonic well-being. Hedonic or emotional well-being includes positive emotions such as happiness and life satisfaction. Eudaimonic well-being includes psychological factors such as meaning, coherence and purpose in life, as well as social factors such as a sense of belonging, integration and contribution. Positive mental health is linked to increased work and social functioning, as well as a decreased health risk and positive behaviors (De Neve et al., 2013).

Although adverse events can damage people's mental health, individuals can also adapt during harsh times. Traumatic experiences sometimes uncover an incredible resilience that can even lead to growth (Linley and Joseph, 2004).

### Protective Factors for Mental Health

During trying times, a number of factors can help individuals survive, manage and adapt. Bassi et al. (2021) found that Italian health workers classified as *thriving* individuals were less likely to have post-traumatic stress (PTSD) during the pandemic. The term thriving denotes the state of fully functioning in mental, physical, and social terms (Su et al., 2014). Thriving includes a range of dynamic factors including, but not limited to, *gratitude* (Emmons et al., 2003; Cheng et al., 2015; Otto et al., 2016), *belonging* (Baumeister and Leary, 1995), *Social- contribution, integration, and actualization* (Provencher and Keyes, 2011), *meaning* (Schueller and Seligman, 2010), *pride* (Tracy and Robins, 2007; Williams and DeSteno, 2008; Fredrickson, 2013), *compassion* (Seppala et al., 2013), and *learning from the situation* (Jenkins and Mostafa, 2015). Broader literature about mental health has highlighted the protective role thriving has in buffering against mental illness (Provencher and Keyes, 2011).

Additional lines of investigations point to the contribution of regular routines to mental health. A recent longitudinal study conducted during the three first months of the pandemic in Germany (Bendau et al., 2020) found that the following factors protected against anxiety and depression: self-efficacy, *normalization of routines*, maintaining social contacts, and knowledge about where to get medical support. Moreira et al. (2021), investigating the same topic during the COVID-19 outbreak in Portugal, found that working (online or in-person) and *physically exercising on a regular basis*, not having previous psychological/physical diseases, not consuming COVID-19 information and doing remote psychotherapy served as protective factors for mental health. These results align with a body of literature that points out that *regularity* has a beneficial effect on mental health (Sano et al., 2017; Murray et al., 2020) in the same way unpredictability in the environment is seen as a potential risk for later mental illness (Glynn and Baram, 2019). Other works showed that regularity of sleep, exercise and social rhythm correlated with improved mental health and well-being (Margraf et al., 2016; Boland et al., 2019; Logan and McClung, 2019).

In addition to person-focused factors, society-directed attitudes can also play a protective role in the context of the pandemic. Although some authors claim that societal trust increases after natural disasters due to the shared need to overcome the event (e.g., Toya and Skidmore, 2014), others maintain that disasters can foster suspicion conspiracy theories about the event (Wilson and Rose, 2014). These society-level outcomes, in turn, impact mental health. O'Hara et al. (2020), for instance, found that in countries that distrusted the government, an increase in policy stringency was associated with men reporting more depression—but no more worries—and women reporting both worries and depression. Thus, *trust in government* can impact well-being (Helliwell and Huang, 2008), and *low perceived efficacy of governmental actions* can reduce mental health (Mækelæ et al., 2020), especially during pandemic times when governments have to impose behavioral restrictions.

Finally, decades of research demonstrate that social connections are vital to the well-being and coping with difficult situations (Sibley et al., 2020). Thus, *social support* and *close and caring relationships* may both help individuals cope with life's adversities, as well as foster growth and development (Feeney and Collins, 2014). Social support is well-known to be a protective factor for mental disorders (Puschner, 2018), including paranoia (Freeman et al., 2011; Crush et al., 2018) and depression (Høifødt et al., 2020).

### Risk Factors for Reduced Mental Health

Numerous risk factors are also present during trying times, having an adverse effect on the physical and mental health of individuals. Bendau et al. (2020) found that anxiety and depression are exacerbated by routine suppression, unhealthy diet, reduced physical activity, increased substance abuse, and a longer daily time thinking about the pandemic Xiong et al. (2020), in turn, found that women, younger people (≤40 years), individuals with chronic/psychiatric illnesses, unemployed, students, and people frequently exposed to COVID-19 news experience more negative impact on their mental health in eight countries (China, Spain, Italy, Iran, USA, Turkey, Nepal, and Denmark). An additional study conducted in China (Guo et al., 2020) has identified, in addition to the factors mentioned above, the following risk factors: reduced income, having family members with chronic diseases, concerns related to COVID-19 infection for themselves/family members, living alone, having family conflicts, having sedentary time per day, and worsened sleep quality. Furthermore, a study conducted in the USA found that fear, worry, and threat were significant predictors of both depressive and anxiety symptomatology, even after controlling social vulnerability measures (Fitzpatrick et al., 2020).

The studies above suggest that excessive worry, catastrophizing thoughts, feeling scared about COVID-19 infection together with other fears, and experiencing a lack of control are all components of a *negative mindset* related to poor mental health. These studies also highlight vulnerability conditions, for example *low socio-economic status* (Link and Phelan, 1995; Reiss, 2013), *low levels of education* (Araya et al., 2003), *presence of financial worries* (Bareket-Bojmel et al., 2020), unemployment (Xiong et al., 2020), or reduced income (Guo et al., 2020), are risk factors for the mental health of specific importance during unpredictable times, such as a pandemic. Finally, the frequent exposure to COVID-19 news (Bendau et al., 2020; Xiong et al., 2020) accompanied by *low perceived efficacy of governmental actions* (Mækelæ et al., 2020) can make people react with suspicion and develop conspiracy theories about it (Wilson and Rose, 2014). According to Uscinski et al. (2020), during the COVID-19 outbreak there has been an increase in irrational beliefs or conspiracy theories, possibly due to decreased social interactions (Graeupner and Coman, 2017), potentially leading to detrimental outcomes for individuals (Bierwiaczonek et al., 2020) and societies alike (Jolley and Paterson, 2020; Romer and Jamieson, 2020).

### The Current Study

Although some of the aforementioned factors are fairly universal, other factors depend to varying degrees on local spatial and temporal contexts. For example, countries vary in their age distribution, levels of trust in the local government, the prevalence of higher education among citizens, and degree of social equality to name a few parameters. Opinion papers have highlighted the potential contribution of psychological science to coping with the pandemic (Arnot et al., 2020; Garfin, 2020; van Bavel et al., 2020) and a couple of reviews (Serafini et al., 2020; Talevi et al., 2020) and empirical papers have investigated the impact of COVID-19 on the mental health of individuals (e.g., Bendau et al., 2020; Martínez et al., 2020). However, only some of these studies about COVID-19's detrimental impact on mental health have been conducted across countries (see e.g., Gobbi et al., 2020; Xiong et al., 2020; Alzueta et al., 2021; Gato et al., 2021).

Several international organizations published first recommendations (e.g., WHO, 2020) highlighting potential risk and protective factors that might assist in the prevention of mental health issues arising from the COVID-19 pandemic. Here we aimed to further characterize the robustness of factors that help maintain mental health during the COVID-19 outbreak through a two-wave observational study conducted across five countries. The goal of this work was two-fold. First, we wanted to determine whether the prolonged disruption of normal life and imposed social restrictions increased general distress and paranoia. Second, we aimed to identify which factors contribute to maintaining mental health during the pandemic. We hypothesized that the negative effects of social restrictions will be attenuated by the presence of protective factors (having high trust in the government/authorities, thriving, exercising, engaging in actions for own and others' well-being, maintaining a regular schedule, and having no financial worries). We further hypothesized that these negative effects will be amplified by the presence of risk factors (having high perceived risk, financial worries, and lower education). Identifying the factors that improve mental health (by reducing general distress and paranoia) can assist governments worldwide in handling the long-term social and economic costs associated with coping with the societal and psychological aspects of a pandemic (Nicola et al., 2020).

## Methods

### Design

The present study used a longitudinal observational design with two waves of data collection (April and July 2020) in a convenience sample composed of participants from five countries: Brazil, Colombia, Germany, Israel and Norway. In mid-April 2020 we launched a survey in seven languages targeting five countries: Norway (Norwegian), Germany (German), Israel (Hebrew and Arabic), Colombia (South American Spanish), Brazil (Brazil-Portuguese). We included these specific countries to allow rapid data collection in an early stage of the pandemic (see Mækelæ et al., 2020). The survey was also available in English in all countries but Israel.

### Recruitment and Participants

The first wave (W1) occurred in mid-April 2020 and was composed of over 2,200 participants. The second wave (W2) happened in July and was composed of over 700 participants who took the follow-up survey. Table 1 reports the sample characteristics for the five countries collected in wave 1.

**Table 1:** Sample demographics, affection and selected daily activities in April.

	**Brazil**	**Colombia**	**Germany**	**Israel**	**Norway**
N_April/N_July	384/86	353/118	273/61	372/77	832/389
Mean age (range)	44 (18–72)	25 (18–72)	46 (19–74)	37 (18–73)	40 (18–74)
Female/male/other	303/80/1	228/122/3	214/58/1	255/116/1	617/213/2
Female/male %	79/21%	65/35%	78/22%	69/31%	74/26%
Urban vs. rural	361 vs. 23	333 vs. 19	155 vs. 113	308 vs. 57	593 vs. 230
in %	94%	94%	57%	83%	71%
Single households (%)	60 (16%)	11 (3%)	60 (22%)	38 (10%)	165 (20%)
Wealth (low-middle-upper)	16-153-202	108-235-8	74-186-7	61-262-37	90-696-31
	4/40%	31/67%	27/68%	16/70%	11/84%
% higher education	87%	76%	58%	52%	85%
**Affected**					
Governmental Quarantine	58%	93%	7%	63%	9%
Self-chosen Quarantine	35%	15%	18%	36%	24%
Social distancing	82%	97%	52%	30%	64%
Has/had COVID-19	3/2	0/0	1/5	1/1	1/4
Family COVID-19	2	10	4	6	6
Essential worker (%)	23 (6%)	4 (1%)	57 (21%)	69 (19%)	174 (21%)
**Daily activities**					
Home office (>2 h) vs. N/A	65 vs. 12%	80 vs. 3%	41 v 39%	62 vs. 15%	59 vs. 22%
Office (>2 h) vs. N/A	14 vs. 68%	6 vs. 85%	31 v 48%	18 vs. 68%	27 vs. 52%
Childcare	34%	15%	37%	45%	51%
Exercising	75%	78%	86%	87%	89%
At least 30 min outside	53%	38%	83%	63%	75%
Watch news > 2 h	39%	13%	27%	25%	22%
Communicating > 2 h	35%	29%	11%	26%	12%

*Wealth is grouped into low by pooling the lowest two self-ratings (belonging to the bottom 10 and 11–30%), middle by pooling the self-rating belonging to 31–60% and 61–90%, upper is self-rated top 10%*.

The inclusion criteria were to have internet access and to be older than 18 years old. The exclusion criteria were to complete the survey in <3 min and to answer fewer than 70% of the items on a scale. Participants were recruited via social media (FB ads) and snowballing. All participants were encouraged to answer all items. Responding took around 15 min in April and around 10 min in July. All participants provided their informed consent and they did not receive any compensation. After answering the first wave, we asked respondents whether they would volunteer to partake in a follow-up in ~2 months later. If they consented they were transferred to a new survey collecting their email addresses.

### Assessments

Our survey included several distinct constructs, some developed anew, e.g., trust scale, thriving and negative mindset scale, others modified from established scales (e.g., CORE-10, CAPE-42, epistemic belief), across four categories. We describe the measures for each category below.

#### COVID-19 Restrictions, Reactions, and Reported Behavior

##### Experienced Restrictions

We asked what outbreak-related impacts the respondent has experienced. Answers included government-issued quarantine, self-determined social quarantine, being an essential worker, and having COVID-19 or have recovered from COVID-19. We measured the experienced restrictions on a nominal scale, with multiple answers possible per participant.

##### Perceived Efficacy of Actions

We measured the perceived efficacy of own, others', and governmental actions on a 5-point Likert scale (0 = don't agree, 4 = fully agree) (Mækelæ et al., 2020). We calculated an average efficacy of action score from these three items. Internal consistency was McDonalds ω = 0.615 in April and ω = 0.592 in July.

##### Timeframe

We asked how long people think the COVID-19 outbreak will last, and how long the governmental-issued restrictions will last. Answer options ranged from 1 to 2 weeks, 2 to 4 weeks, 1 to 3 months, 3 to 6 months, 6 months to 1 year, 1 to 2 years, to forever.

##### Protective Behavior

We asked how often on a usual day each of 17 activities was done. Answer options ranged from <30 min, 30 min to 2 h, 2 to 5 h, more than 5 h, and not applicable. Activities were: (1) working at one's regular workplace, (2) working from home, (3) going out of the house, (4) exercising, (5) DIY activities, (6) doing chores around the house, (7) providing emotional support to somebody, (8) caring for kids, (9) watching news, (10) watching movies, (11) playing, (12) meditating, (13) praying or other religious activities, (14) talking to or messaging with friends and family, (15) communicating with friends and family, (16) helping friends and family, (17) procrastinating. Not all activities were applicable when in quarantine, e.g., working outside the house and not everybody may have to care for children.

##### Regularity

We measured the extent to which respondents maintained a regular schedule on a three-item scale; (a) keeping a regular schedule, (b) eating regularly and (c) sleeping at a regular time during the outbreak, measured on a 5-point Likert scale (0 = no regularity, to 4 = high regularity). We calculated an average score and the scale's reliability had ω = 0.749 in April and ω = 0.804 in July.

##### Finances

We asked whether the outbreak changed the financial circumstances of the respondent. Answer options were; yes, lost income; No; don't want to answer; and yes, increased income. We also asked about their financial worries, which was measured on a VAS ranging from not worried at all (coded as 0) to extremely worried (coded as 100).

#### COVID-19 Psychological Measures

##### Perceived Risk

We included five items to ask about perceived risk of (a) contracting COVID-19 within the next week, (b) within the next 2 months, (c) getting seriously ill if contracted; (d) chance of having COVID-19 and infecting others (asymptomatic spreader), and (e) chance of dying during the outbreak. The first three items are identical to the scale used by Mækelæ et al. (2020). We used a visual analog scale (VAS) ranging from 0 (no risk) to 100 (certainty). We calculated an average perceived risk score and the scale's reliability had ω = 0.738 in April and ω = 0.783 in July.

##### Trust in Authorities

*Overreaction.* We asked respondents whether their country does enough to fight the outbreak. Answer options were: “does enough,” “don't know,” “does not enough,” and “overreacting” (Mækelæ et al., 2020). Furthermore, if they chose overreacting participants were asked three additional items; (a) overreacting because the virus is not that dangerous, (b) unreasonable restricting my personal freedom, (c) personal and financial costs are greater than the threat by the virus. This was measured on a 5-point Likert scale ranging from strongly disagree (1) to strongly agree (5).

*Trust.* We used 8 items, newly created, to ask about trust, belief and confidence in government, the healthcare system and researchers/science on a 5-point Likert scale from 0 (don't agree) to 4 (fully agree). We used the average score and the scale's reliability had ω = 0.936 in April and ω = 0.944 in July.

*Conspiracy Score.* We asked how much respondents endorsed different conspiracy theories such as “The virus is part of a Chinese biological weapons program.” We also presented three factual statements, e.g., “the virus belongs to the SARS family.” Responses were scored on a VAS from 0 = not true at all to 100 = absolutely true (Mækelæ et al., 2020). We calculated a difference score between belief in conspiracy theories and knowledge. A positive score indicates endorsement of conspiracy theories. McDonald's ω was 0.734.

*Thriving.* We used a newly created Thriving Scale based on research into optimal human functioning and positive psychology (Maslow, 1965; Antonovsky, 1979; Ryff and Singer, 1996; Ryan and Deci, 2000; Keyes, 2005; Diener et al., 2009; Seligman, 2011). The scale was adapted to assess important factors for thriving in the situation of a large scale crisis, and items were created similar to our ESM studies (Lüdtke et al., 2021). It has eight items on a 5-point Likert scale ranging from 0 (don't agree) to 4 (strongly agree). Items probed social contribution and finding meaning (“Helping and contributing in this time feels meaningful”), Sense of belonging (“This situation makes me feel like a part of a larger community”), Pride and social actualization (“I am proud of how my community is responding to this crisis”), Gratitude (“In this situation I still have so much to be grateful for”), Compassion (“I am moved by others suffering and I want to help”), social integration and common purpose (“We all need to work together in this situation”), belief in growth and learning (“We can learn a lot from this situation”), and social norms (“I follow the guidelines”). We calculated an average score. McDonalds ω was 0.817 in April and ω was 0.755 in July.

*Negative Mindset.* We used a newly created Negative Mindset Scale with six items on a 5-point Likert scale. The items were; excessive worry (“I am very worried about the outbreak”), catastrophizing thoughts (“I fear that the infrastructure will break down,” “I feel humanity will never be the same after this outbreak”), experiencing a lack of control (“The uncertainty of this time scares me,” “I feel we can control the outbreak” (reverse scored), and feeling scared (“I am scared of the outbreak”). We calculated an average score. Internal consistency was ω = 0.777 in April and ω = 0.775 in July.

*Epistemic Belief.* We adapted two items from the epistemic-aleatory uncertainty scale (Ülkümen et al., 2016); (a) The COVID-19 outbreak has an element of randomness; (b) The COVID-19 outbreak becomes more predictable with additional knowledge or skills. Scoring was on a VAS from 0 = not at all true to 100 = absolutely true. We calculated a difference score of epistemic and aleatory uncertainty, i.e., average VAS score for (b) and (a). A positive score might indicate a more scientific thinking style.

*General Distress.* We measured global distress with 9 items from the Clinical Outcomes in Routine Evaluation (CORE-10) (Connell et al., 2007). As advised by the ethical committee, we omitted the “I made plans to end my life” item as a high score on this item mandates counseling. The scale ranged from not at all (0) to most or all of the time (4). We calculated a sum score for the CORE-9. Internal consistency was ω = 0.86 in April and ω = 0.859 in July.

*Paranoia.* We measured paranoid thoughts with 10 items on persecutory and grandiose delusions, and on anomalous perceptions through the Brief 10-Item Community Assessment of Psychic Experiences-Positive Scale (Brief CAPE-P10, items: 2, 6, 7, 10, 13, 22, 32, 33, 42) (Stefanis et al., 2002). Answer options were from never (0) to nearly always (3). We calculated an average score. The Brief CAPE-P10 score had ω = 0.833 in April and ω = 0.812 in July.

*Demographics.* We asked for age, gender, country of residency, education (five steps from <8 years of schooling coded as 0 to Master/PhD education coded as 4), SES (asking in five steps from bottom 10% to top 10%), people (separate children) living in the household, living space (asking from <10 square meters to more than 120 square meters). We also asked coarsely for political affiliation, i.e., “In political matters, people often talk of ‘the left’ and ‘the right’. How would you place your views on this scale, generally speaking?” in all but the Norwegian and German survey.

The same items were used in the July survey with the exception of omitting the knowledge about the virus scale, i.e., there was no conspiracy score. We also asked for fewer demographic items in the July survey. Presentation of the order of items within a scale were randomized.

### Statistical Analysis

To test which factors predict general distress (hypothesis 1) we conducted a linear mixed model with the general distress score (CORE-9) as the outcome, country as a random effect and thriving, regularity, trust, financial worry, paranoia, negative mindset, perceived risk, perceived efficacy of actions, gender, and education as the predictors. Survey distribution time (April or July) was entered as a fixed effect.

To address hypothesis 2 we run a linear mixed model for the paranoia score (CAPE-P10) as the dependent variable and as fixed effects: thriving, regularity, trust, financial worry, negative mindset, general distress, perceived risk, perceived efficacy of actions, gender and education. Country was entered as a random effect and wave as fixed effect. We also run a linear mixed model with the same predictors complemented with the conspiracy score for the April data.

Since we expected a positive relationship between general distress and paranoia (Sun et al., 2019; Mækelæ et al., 2020), we applied mediation analysis with general distress being the predictor, paranoia score the outcome and thriving, regularity, trust, negative mindset, perceived risk and perceived efficacy as mediators.

To examine the predictors of satisfaction and dissatisfaction with the actions taken to counteract the outbreak in one's country (hypothesis 3) we conducted a generalized mixed model with satisfaction as the binary outcome variable with the following predictors: thriving, regularity, trust, financial worry, conspiracy score, paranoia, negative mindset, general distress, perceived risk, efficacy of actions, gender and education. Country was included as a random effect. For comparing April and July (fixed effect) the predictor conspiracy score was omitted.

To examine the relationship between usual day activities and general distress we performed correlation analyses and compared April and July correlations using z transformation.

We used frequentist analysis and a significance criterion of *p* < 0.05 for the two mixed models and for the logistic regression (pre-registered hypotheses). All predictors and outcomes were centered. For the exploratory analysis we corrected for multiple testing. We used Jamovi (The Jamovi Project, 2021) and R (R Core Team, 2017) for data analysis and visualization. Results without country as a predictor are reported in the Supplementary Material (SOM).

The analyses in this study were formally pre-registered. However, the analyses presented below deviate from the pre-registration and are only conceptually similar, as our statistical approach focused on longitudinal outcomes and required us to collect over 1,000 responses in July. In reality, we were able to collect only 731 valid responses in July.

### Power Analysis

There is considerable variation in the recommendation of required samples for multiple and hierarchical regression analysis. We follow a rule of thumb for multiple regression (Brysbaert, 2019), i.e., 100 participants plus another 100 for every predictor. Since we had up to 11 predictors, we aimed to recruit at least *N* = 1,200 participants to partake in each survey.

## Results

Over 2,500 participants answered our April survey, with 2,214 respondents from our five target countries. The remaining respondents were from Sweden (*n* = 37), the US (*n* = 85), UK (*n* = 21), Canada (*n* = 15), France (*n* = 10), Austria (*n* = 8), Denmark (*n* = 6) and the remaining from over 25 other countries. In July 844 participated in the follow-up survey, with 731 from the five target countries and the remaining respondents were from the US (*n* = 33), Austria (*n* = 7) and over 15 other countries.

Our sample was well-educated, over 70% female, most identified themselves as belonging to the middle class, and very few had an infection with SARS-CoV-2 (for details see Table 1). The sample in July was similar in age [*F*_(1, 2,932)_ = 4.94, *p* = 0.026, $\eta_{\text{p}}^{2}$ = 0.002], gender (χ^2^ = 1.37, *p* = 0.504), education [*F*_(1, 2,655)_ = 0.078, *p* = 0.78, $\eta_{\text{p}}^{2}$ < 0.001] and reported SES [*F*_(1, 2,477)_ = 1.53, *p* = 0.0216, $\eta_{\text{p}}^{2}$ = 0.001], suggesting no systematic attrition.

The countries differed markedly in the proportion of participants stating that their country overreacted, 46.5% of German respondents said so, followed by 15% of participants from Israel, 4% from Norway, 2% from Brazil and <1% from Colombia. Those participants felt that the costs of a lockdown are too high and their personal freedom was too restricted. Only half of them stated that the virus is harmless.

**Figure 1:**
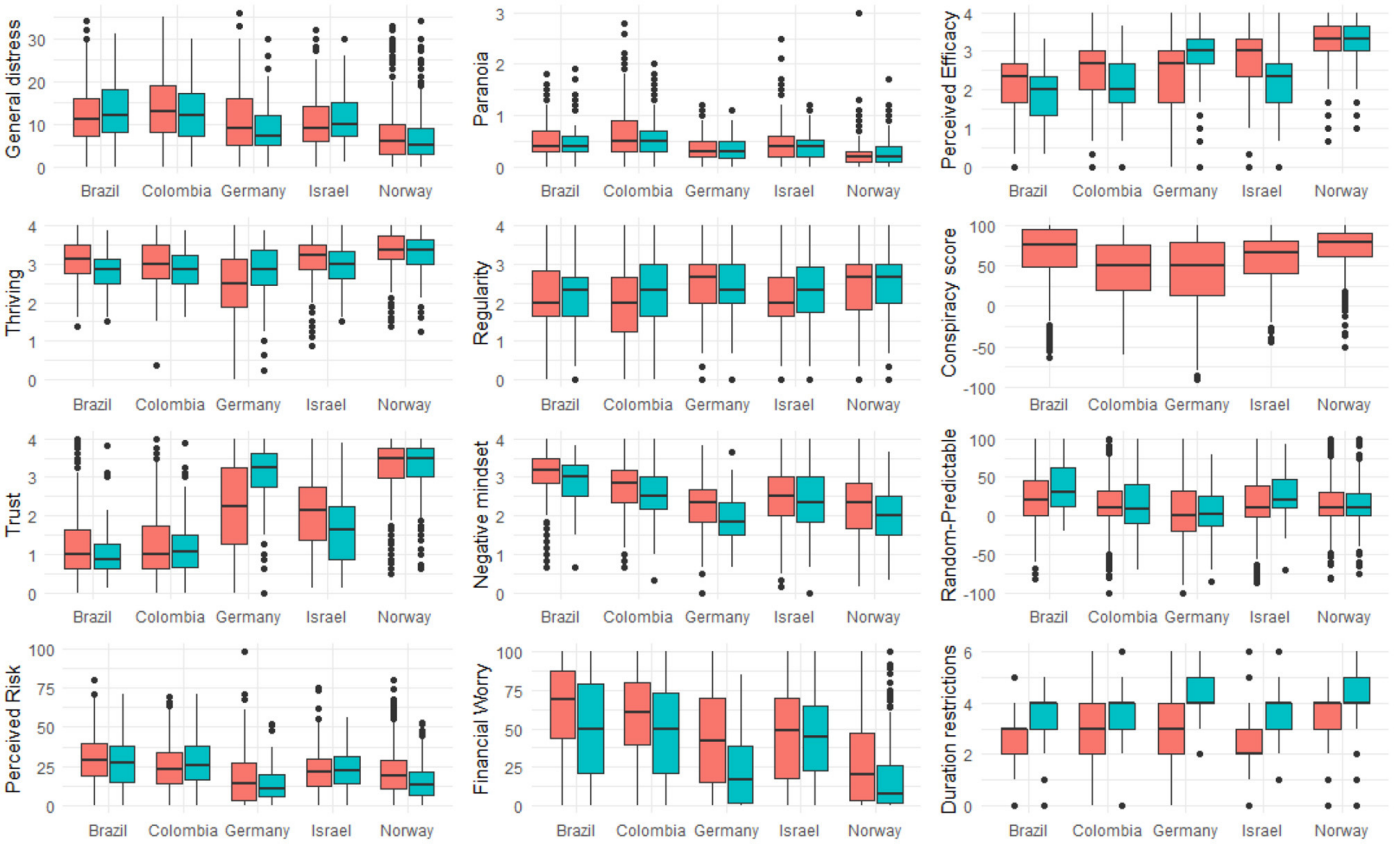
Boxplots indicating the median, second and third quartiles and outliers for the dependent and independent measures included in our study. Red = April data, Blue = July data. No data are available for conspiracy theories in July as we omitted this scale. For statistical details see Supplementary Material, page 3ff.

As can be seen in Figure 1 from April to July there was no change in general distress and paranoia [statistical details, reporting difference between the countries are reported in the Supplementary Material (SOM)]. Thriving decreased in four of the five countries from April to July. Regularity, on the other hand, increased slightly from April to July. As for trust in their authorities, overall trust did not change from April to July. On average, there was a reduction in negative mindset from April to July whereas the predictability rating of the pandemic slightly increased from April to July. Across all five countries, perceived risk of COVID-19 did not change from April to July. Financial worries, on average, reduced from April to July. Regarding the expected duration of the restrictions, across all countries participants expected longer lasting restrictions when asked again in July.

### Protective and Risk Factors for General Distress

**Figure 2:**
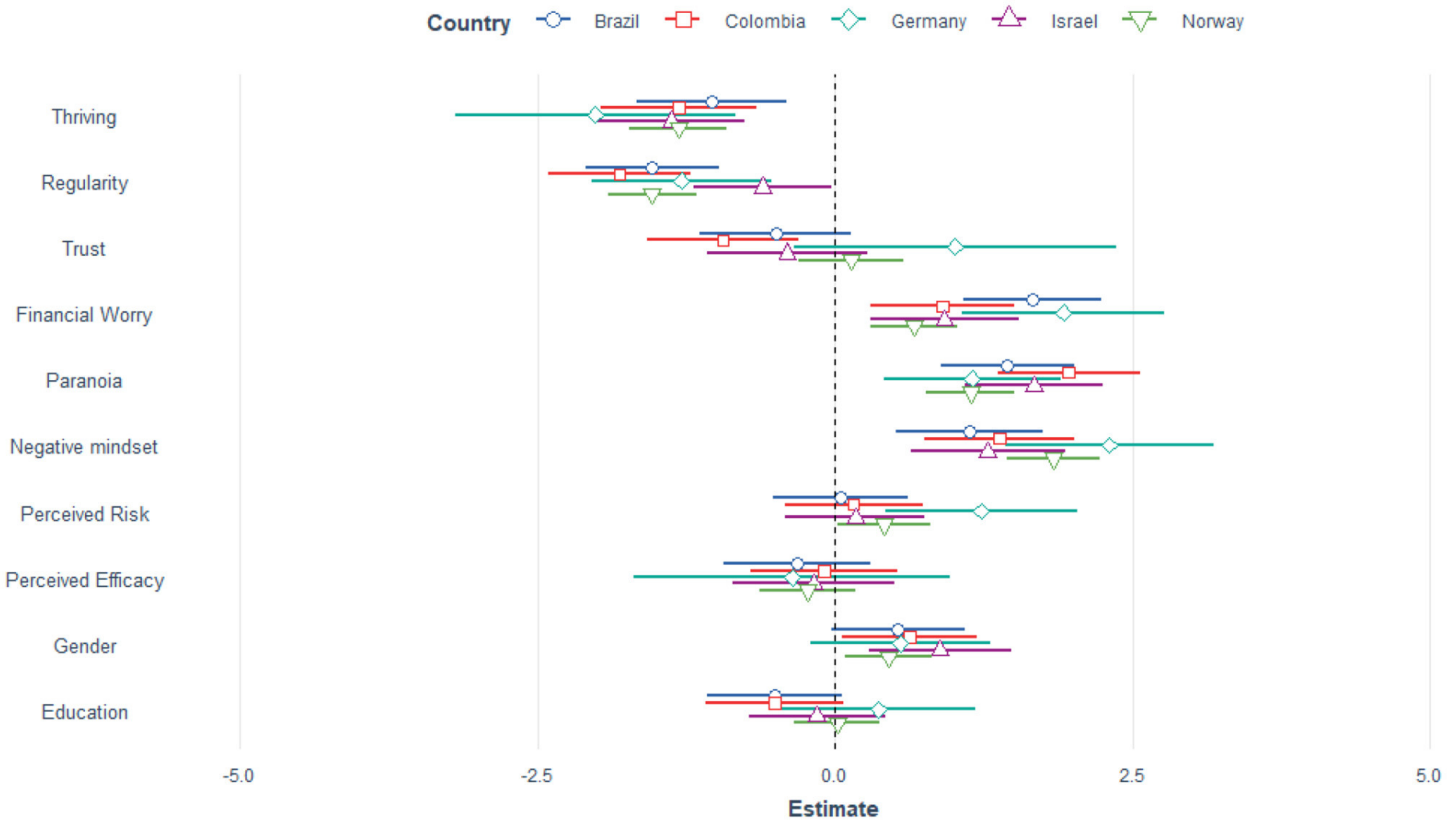
Estimates for the 10 predictors of general distress per country, April data. Gender is coded as male = 0, 1 = female, 2 = other.

**Figure 3:**
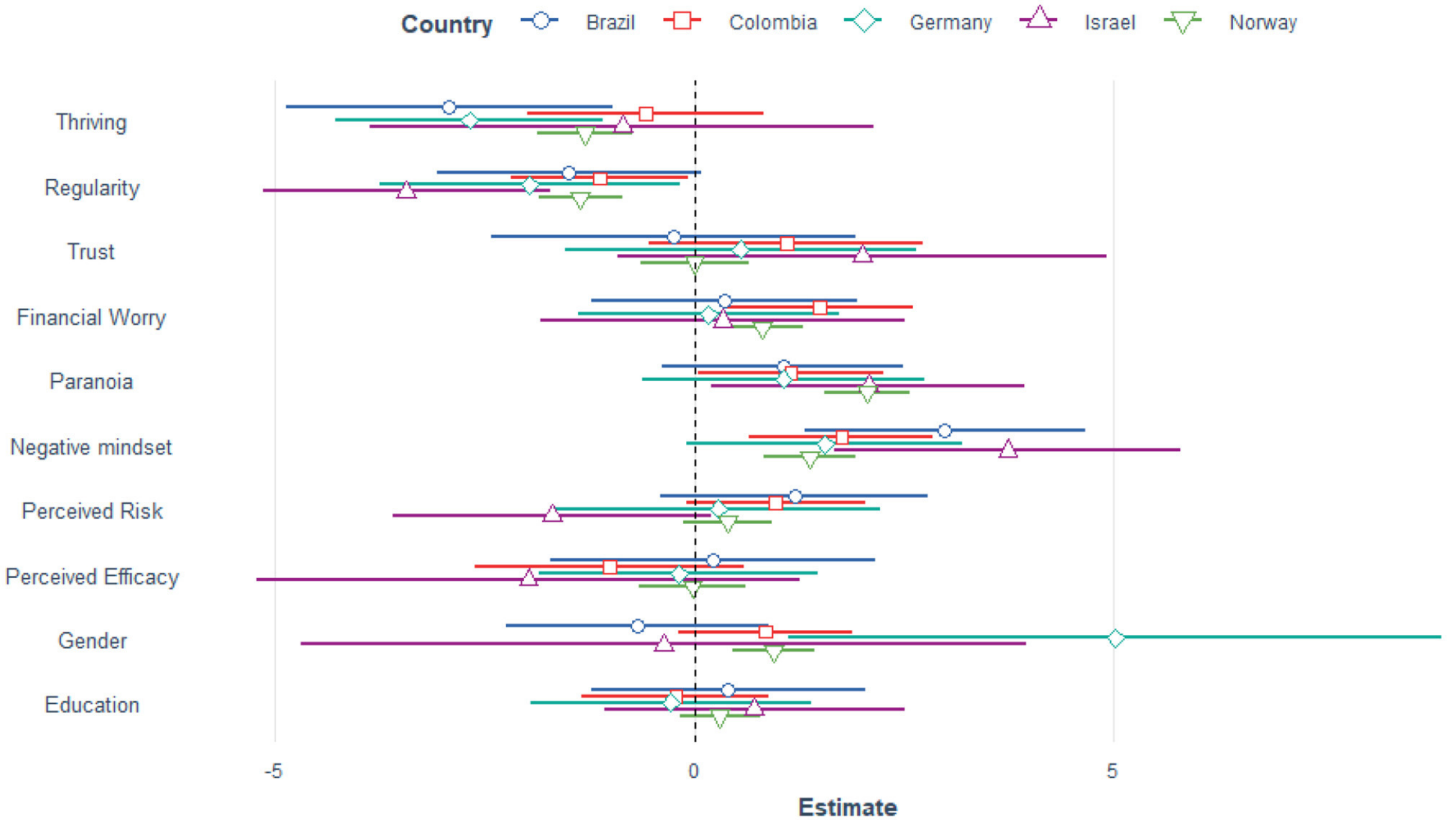
Estimates for the 10 predictors of general distress per country, July data. As can be seen, the difference between the months and between the countries is smaller than between the predictors. Gender is coded as male = 0, 1 = female, 2 = other.

The linear mixed model testing our first hypothesis explained 44.7% of the variance. There was no significant change in distress from April (Mean= 9.73, SD = 0.291) to July (Mean = 10.11, SD = 0.395). Perceived efficacy of governmental reactions and trust in authorities were not significant predictors of general distress. Thriving and maintaining a regular schedule demonstrated protective qualities, i.e., higher scores yielded lower general distress. A negative mindset, paranoia, high perceived COVID-19 infection risk, financial worries and being female predicted more distress. The random effect of country was significant (LRT^1^ = 9.27, *p* = 0.002). Table 2 reports the estimates of the mixed model and Figures 2, 3 shows the estimates per country and for the April and July survey, respectively.

**Table 2:** Fixed effects parameter estimates for general distress.

			**95% confidence interval**				
**Predictor**	**Estimate**	**SE**	**Lower**	**Upper**	**df**	***t***	***p***
(Intercept)	9.918	0.311	9.309	10.527	4.86	31.929	<0.001
Thriving	−2.071	0.206	−2.474	−1.667	1,386.07	−10.05	<0.001
Regular schedules	−1.5	0.119	−1.733	−1.267	2,496.67	−12.643	<0.001
Trust in authorities	−0.225	0.151	−0.521	0.07	567.56	−1.494	0.136
Financial worry	0.034	0.004	0.027	0.042	2,464.44	8.772	<0.001
Paranoia	4.69	0.35	4.003	5.376	2,349.32	13.394	<0.001
Negative mindset	2.103	0.167	1.777	2.429	2,391.18	12.629	<0.001
COVID-19 risk	0.031	0.008	0.016	0.047	2,497.91	3.99	<0.001
Perceived efficacy	−0.032	0.178	−0.382	0.317	2,349.51	−0.182	0.855
Gender	1.303	0.238	0.837	1.769	2,491.69	5.478	<0.001
Education	−0.221	0.126	−0.469	0.026	1,890.57	−1.753	0.08
T2July - T1April	0.38	0.308	−0.224	0.984	2,418.79	1.234	0.217

### Protective and Risk Factors for Paranoia

**Figure 4:**
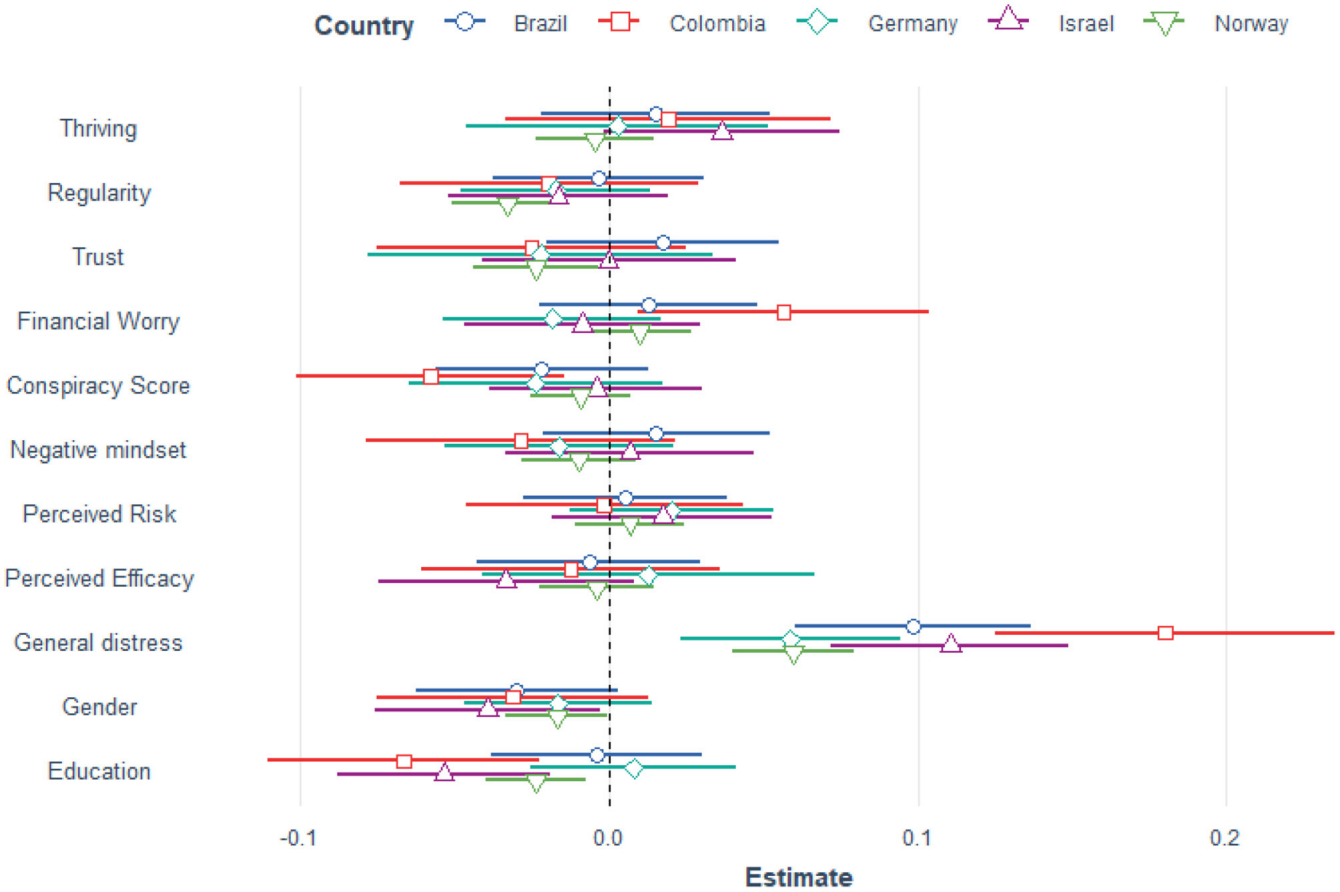
Estimates for paranoia per country, April data.

The linear mixed model for paranoia and for April and July explained 25.5% of the variance. The fixed effect of wave was small, *F*_(1, 2,498)_ = 4.042, *p* = 0.045. We therefore report the model for April only but including the conspiracy score as predictor. The mixed model for paranoia in April explained 24.5% of the variance. The paranoia score differed by country (LRT = 75.4, *p* < 0.001). The less regularity and trust respondents reported, and the lower their level of education, the more paranoia they experienced. Being male was also associated with paranoia, so was a higher conspiracy score and general distress. Financial worries, negative mindset, perceived risk, perceived efficacy and trust were not associated with more paranoia. Table 3 reports the estimates and Figure 4 shows that there is more variation between the indices than the countries.

**Table 3:** Fixed effects parameter estimates for paranoia.

			**95% confidence interval**				
**Predictor**	**Estimate**	**SE**	**Lower**	**Upper**	**df**	***t***	***p***
(Intercept)	0.415	0.044	0.329	0.5	4.09	9.478	<0.001
Thriving	0.008	0.012	−0.015	0.031	2,475.05	0.699	0.485
Regular schedules	−0.024	0.007	−0.037	−0.011	2,495.66	−3.572	<0.001
Trust in authorities	−0.012	0.009	−0.029	0.005	2,348.3	−1.41	0.159
Financial worry	0.0004	0.0002	−0.0001	0.001	2,497.58	1.633	0.103
Negative mindset	−0.008	0.009	−0.027	0.011	2,497.99	−0.85	0.396
General distress	0.014	0.001	0.012	0.016	2,495.58	13.299	<0.001
COVID-19 risk	0.001	0.0004	0.0002	0.002	2,495.19	2.49	0.013
Perceived efficacy	−0.02	0.01	−0.04	−0.001	2,497.99	−2.063	0.039
Gender	−0.053	0.013	−0.079	−0.027	2,496.78	−4.055	<0.001
Education	−0.032	0.007	−0.046	−0.019	2,493.36	−4.612	<0.001
T2July - T1April	0.034	0.017	8.55E-04	0.067	2,497.64	2.01	0.045

We found the same pattern of results when analyzing data from April and July separately, using all data irrespective of country. Thriving and regularity were protective whereas paranoia, negative mindset, financial worry and being female were risk factors for general distress (see Supplementary Material for details). Similarly, general distress was a significant predictor for paranoia. The overall pattern for general distress and paranoia emerged also when using the longitudinal subset (*n* = 525), i.e., participants who took part both in April and July (see Supplementary Material for details, page 20).

### Which Factors Mediate the Relationship Between Distress and Paranoia?

As Figures 2–4 show there is a positive relationship between general distress and paranoia. We therefore investigated (using the April data) whether any of the protective and risk factors could mediate the relationship, i.e., would there be a significant indirect effect. Mediators were thriving, regularity, trust, negative mindset, perceived risk and perceived efficacy. The direct effect between distress and paranoia explained 98% of the variance whereas the indirect effect of thriving explained only 2%, Z = 1.75, *p* = 0.079. Similarly a negative mindset did not mediate the relationship, 2.68%, Z = 1.65, *p* = 0.1. On the other hand regularity significantly mediated the relationship between distress and paranoia, 9.27%, Z = 5.03, *p* < 0.001, so did trust: 17.1%, Z = 9.27, *p* < 0.001, perceived risk: 2.51%, Z = 2.62, *p* = 0.009 and perceived efficacy: 7.46%, Z = 5.86, *p* < 0.001. Thus, a more regular schedule, higher trust in authorities, lower perceived risk and higher perceived efficacy of actions reduced the association between distress and paranoia.

### What Characterizes Those Who Think Their Country Overreacted?

Finally, our third hypothesis that predicted more distress and paranoia among those who think that their country overreacted was not confirmed. Firstly, the relative number of participants stating that their country overreacted was lower in July than in April [estimate = −4.42, exp(B) = 0.012, z = −4.84, *p* < 0.001]. Secondly, applying generalized mixed model (logit link function, country as cluster variable/random intercept) for the April data (Table 4), dissatisfied participants had a significant lower score on thriving (χ^2^ = 21.05, *p* < 0.001), trust in authorities (χ^2^ = 17.11, *p* < 0.001) and perceived efficacy (χ^2^ = 10.09, *p* < 0.001), but also demonstrated less characteristics of a negative mindset (χ^2^ = 27.35, *p* < 0.001) and endorsed conspiracy theories more (χ^2^ = 36.73, *p* < 0.001). They showed no differences in general distress, financial worries, regularity, education, gender or paranoia. Their perceived risk was somewhat higher but not significantly so. Overall the model explained 68.5% of the variance (*R*^2^ conditional). Applying a similar model without the conspiracy score but with wave as fixed effect yielded very similar results, i.e., thriving, trust in authorities, perceived efficacy and negative mindset were significant predictors. In addition, lower perceived risk significantly predicted dissatisfaction with the governmental response [estimate = −0.02, exp(B) = 0.978, z = −3.102, *p* = 0.002].

**Table 4:** Fixed effects parameter estimates for satisfaction.

				**95% Exp(B) Confidence Interval**			
**Predictor**	**Estimate**	**SE**	**exp(B)**	**Lower**	**Upper**	**z**	***p***
(Intercept)	−3.786	0.895	0.023	0.004	0.131	−4.229	<0.001
Thriving	−0.830	0.181	0.436	0.306	0.621	−4.587	<0.001
regular schedules	−0.032	0.115	0.968	0.774	1.212	−0.281	0.778
Trust in authorities	−0.59	0.143	0.555	0.419	0.733	−4.137	<0.001
Financial worry	0.003	0.004	1.003	0.996	1.010	0.824	0.410
Conspiracy score	−0.019	0.003	0.982	0.976	0.988	−6.061	<0.001
Paranoia	0.245	0.348	1.278	0.647	2.526	0.705	0.481
Negative mindset	−0.881	0.168	0.415	0.298	0.577	−5.230	<0.001
General distress	−0.019	0.019	0.981	0.945	1.018	−1.009	0.313
Risk	−0.015	0.007	0.985	0.971	0.999	−2.050	0.040
Perceived efficacy	−0.545	0.171	0.58	0.415	0.812	−3.177	0.001
Gender	−0.335	0.227	0.716	0.459	1.116	−1.477	0.140
Education	0.028	0.112	1.028	0.826	1.280	0.248	0.804

### Comparison to March Data

**Figure 5:**
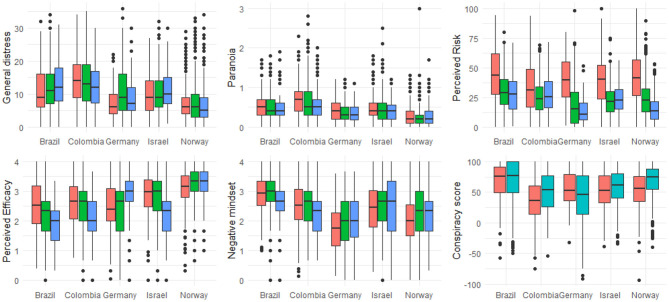
Comparison to our March data. Paranoia and perceived risk declined from March to July. For more details, please see the Supplementary Material.

In addition to the data described so far, we have also collected data in March 2020, investigating the perceived efficacy of COVID-19 restrictions and how those affect mental health (Sun et al., 2019; Mækelæ et al., 2020). The following will capitalize on similarities in the collected measures to cross-sectionally compare general distress, paranoia, conspiracy score and perceived risk, four measures that were identical (distress and paranoia) or had overlapping items (conspiracy score, perceived risk) in all three surveys, see Figure 5.

Distress increased from March to April, *F*_(2, 4,671)_ = 11.851, *p* < 0.001, $\eta_{\text{p}}^{2}$ = 0.004. Paranoia, on the other hand, decreased in all countries from March to April, *F*_(2, 4,677)_ = 16.189, *p* < 0.001, $\eta_{\text{p}}^{2}$ = 0.006. There was a large reduction in perceived risk from March to April and an even greater reduction in July, *F*_(2, 4,656)_ = 359.994, *p* < 0.001, $\eta_{\text{p}}^{2}$ = 0.129. Finally, we compared the conspiracy score in March with April, and this score increased, *F*_(1, 3,891)_ = 11.981, *p* < 0.001, $\eta_{\text{p}}^{2}$ = 0.003, indicating that in April participants were more likely to endorse Covid-19 related conspiracy theories. Details by country and the interactions can be found in the Supplementary Material, page 21ff.

### Exploratory Analysis of Usual Day Activities

Participants were asked to indicate how much time on a usual day they engage in each of 17 activities. Apart from procrastination we assumed that all other activities are either neutral (e.g., child care) or beneficial for mental health (being outdoors). Briefly, we found that procrastinators had a higher score on general distress than participants who spent no or little time procrastinating (ρ = 0.249, *p* < 0.001). General distress was also lower among those who exercised (ρ = −0.131, *p* < 0.001) and could and did go outdoors (ρ = −0.107, *p* < 0.001). Details are reported in the Supplementary Material, page 30.

## Discussion

This study aimed to identify resilience factors protecting from distress and paranoia during the 2020 COVID-19 outbreak. We collected data from more than 2,000 participants in 5 countries (Norway, Germany, Brazil, Columbia, and Israel) in two waves in April and July. We found that the prolonged disruption of normal life and imposed social restrictions did not lead to a gradual increase across time in general distress and paranoia. Compared to pandemic-onset levels in March, paranoia decreased, though distress in April was higher than in March. Furthermore, we identified protective factors that contributed to reducing general distress and paranoia during the pandemic. Our data show a beneficial effect of thriving and maintaining a regular schedule, with little to no influence of demographic factors such as gender, age and education. As predicted, financial concerns increased distress and paranoia. In what follows, we provide a detailed overview of our main findings and their potential implications.

As far as distress and paranoia are concerned, we found no change between April and July. Even though the various countermeasures enacted by governments led to a prolonged disruption of normal life, distress and paranoia did not gradually increase. In fact, paranoia levels even decreased compared to data collected in March (Mækelæ et al., 2020). Rather, our results indicate that the majority of respondents in our sample grew accustomed to the changed circumstances and may have even perceived them as “The new normal.” Supporting this interpretation, our participants indicated in July that they can better maintain a regular schedule (regularity), rated the pandemic as more predictable, and predicted it will last longer compared to the April ratings. In addition, both protective and risk factors for distress exerted decreased influence in July compared to April. It is possible that regularity, predictability and reduced uncertainty had offset the negative impact of the demanding nature of dealing with a pandemic. Increased regularity and predictability may have assisted individuals to perceive the pandemic as more manageable and to better arrange their resources (Lazarus and Folkman, 1974). This is consistent with Antonovsky's (1979) work showing that resilience is enhanced when an event is appraised as comprehensible, manageable and meaningful. Such adaptation might also benefit from self-efficacy (Southwick and Charney, 2012). Already in our March sample, we saw that high perceived efficacy, including own actions, was associated with a feeling of controlling the outbreak (Mækelæ et al., 2020). Together with high levels of social support and physical exercising that may result from regularity, self-efficacy leads to active problem-focused coping and reduce stress levels (Taylor and Stanton, 2007; Southwick and Charney, 2012). Overall, our data show that people may adapt to a demanding situation once it is perceived as predictable and manageable.

Adaptation is a core tenet of human behavior. Within the field of happiness economics, it is known that individuals adapt to both prosperity and to adversity and return to their natural levels of happiness (Carol, 2009; Simchon et al., 2020). Furthermore, people are better at adapting to an unpleasant certainty than they are to uncertainty. A long-term study found a reduction or return to normal levels of anxiety among people in isolation during MERS (Jeong et al., 2016). Studies also have shown reductions in worry (Bendau et al., 2020; Varga et al., 2021) and a slight increase in happiness during the late phase of the COVID-19 lockdown in April 2020 (Stieger et al., 2021). Similarly, a recent study (Bendau et al., 2021) found that COVID-19-related fear, anxiety, and depressive symptoms decreased on average over time (March to June), again showing that most people grow accustomed to challenging new situation over time. Our investigation joins these studies in showing that as far as explicit manifestations of mental well-being go, humans display high adaptability to adverse events.

At the beginning of the pandemic in March, the perceived risk of contagion was higher in the five countries compared to the perceived risk in April (Mækelæ et al., 2020). Perceived risk of contagion across all five countries did not change from April to July, and the perceived risk in July was lower than the perceived risk from March. As perceived risk mediated the relationship between distress and paranoia, fostering valid estimations of perceived risk might assist in attenuating detrimental mental health-related outcomes. Further, risk communication should exploit graphical, verbal and numerical formats to nurture realistic perceived risks (Engeset et al., 2018; van der Bles et al., 2019).

In this study, we used a newly created thriving scale based on research into optimal human functioning and positive psychology (Maslow, 1965; Antonovsky, 1979; Ryff and Singer, 1996; Ryan and Deci, 2000; Keyes, 2005; Diener et al., 2009; Seligman, 2011). The scale had good reliability and its goal was not to measure the minimization of loss but instead a positive response to challenges (O'Leary and Ickovics, 1995). Participants who scored high on thriving, reported less general distress, and were more satisfied with how their country reacted to the outbreak. Our finding suggests that an individual's ability to create meaning and a sense of belonging in a challenging situation, as well as finding opportunities for growth and learning, can act as a buffer against distress. Thriving may benefit from social contribution and actualization, as well as pro-social emotions such as gratitude, compassion, and pride (Tracy and Robins, 2007; Williams and DeSteno, 2008; Schueller and Seligman, 2010; Provencher and Keyes, 2011; Cheng et al., 2015; Otto et al., 2016). Indeed, the interpersonal aspect may be substantial and could act as a catalyst for thriving (Feeney and Collins, 2014). Similarly, social support, mastery and optimism are found to be coping resources for stressful events (Taylor and Stanton, 2007; Southwick and Charney, 2012).

Regularity of sleep, eating and daily schedule was greatly beneficial for reducing both general distress and paranoia. These findings are consistent with previous work showing that regularity of sleep, exercise and social rhythm is linked to improved mental health and well-being (Grandin et al., 2006; Margraf et al., 2016; Boland et al., 2019; Logan and McClung, 2019). Maintaining regularity is especially important if circadian rhythms and related routine social cues (zeitgebers) are hampered during the pandemic, and can be a potential target for intervention (Murray et al., 2020).

We did not find that trust in government was a protective factor against general distress and paranoia. Notably, previous investigations have highlighted trust as an important factor in preserving mental well-being (Helliwell and Huang, 2008). This is true also in the specific context of the early phase of the Covid-19 pandemic (Bäuerle et al., 2020; Harris and Sandal, 2020; Jovančević and Milićevi, 2020; Mækelæ et al., 2020; Paolini et al., 2020). The countries included in the present study differed significantly on trust in authorities and distress with Brazilians and Colombians reporting lower trust and higher distress than the citizens of other countries (see Figure 1 and Supplementary Material), potentially indicating the specific socio-political circumstances in each country that might have led to different contributions of trust across these countries.

In the domain of risk factors, we found that excessive worry, catastrophizing thoughts, feeling scared and experiencing a lack of control, all components of the negative mindset scale, were consistently related to increased general distress. Catastrophizing, broadly conceived as an exaggerated negative “mental set,” is associated with high levels of situational depression, anxiety, anger, and sadness. These transient subclinical states of emotional distress could be the vehicle through which catastrophizing impacts on pain mental health (Sullivan et al., 2001). This is in line with a breadth of recent research on worry and mental health (Freeman et al., 2011; Sun et al., 2019; Bendau et al., 2020; Lüdtke et al., 2021). Similarly, the similar levels of general distress compared to March accompanied by the reduced perceived risk of contagion suggest that financial and additional worries (measured by the negative mindset scale) contribute to general distress after the initial uncertain phase of the pandemic has passed.

Our results are also in line with previously reported strong relationships between irrational beliefs and distress (Vîslă et al., 2016). Indeed, recent research links paranoia and delusions to heightened perceived volatility (Deserno et al., 2020; Kreis et al., 2021). A reduced feeling of control, in combination with reduced regularity and less trust, might explain why a small proportion of respondents endorse conspiracy theories and paranoid ideations (Graeupner and Coman, 2017; Bierwiaczonek et al., 2020; Jolley and Paterson, 2020; Jovančević and Milićevi, 2020; Romer and Jamieson, 2020; Uscinski et al., 2020).

### Limitations

Our study is a convenience sample, biased toward educated and female respondents and only very few have had a SARS-CoV-2 infection or a close family member who have had it. Particularly in Brazil, a country with high social inequality and unequal access to the internet (Silva et al., 2020), our sample is mostly from the middle class.

To ensure a high completion rate of the survey we limited the number of protective and risk factors. We did not ask about sleep quality, though sleep quality is beneficial for mental health, e.g., Lüdtke et al. (2021). We used an abbreviated version of the CAPE positive scale and the short form of the clinical outcome (CORE) measure. We also did not ask about protective behavior, or used scales to measure anxiety.

The different scales implemented in this study generally had good internal consistency, both across and within countries (see Supplementary Material for details). One notable exception pertains to the perceived efficacy of actions scale, with internal consistency around 0.6. Accordingly, the lack of effect of this scale on distress and paranoia, as well as its role in mediating the relationship between distress and paranoia, should be interpreted with caution.

The selection of the five countries is due to the researcher's location and access to those data. Our focus was not in comparing those five countries, quite the opposite. We set out to find similarities despite known economic and cultural differences.

### Conclusions

Our study exploited the unprecedented opportunity to measure general distress and paranoia in the general population across five countries varying in their socio-economy during the early phase of the COVID-19 outbreak. Despite large differences in the countries' welfare system and handling of the pandemic, we found the same psychological factors being associated with distress and paranoia. Maintaining a regular schedule was greatly beneficial for both general distress and retaining low paranoia. Across all countries, thriving was highly beneficial for reducing general distress, whereas financial worries and a negative mindset were associated with increased general distress. In summary, our data show the remarkably adaptive and resilient nature of human beings as they grow accustomed to new situations when they have a supportive community and a sense of meaning in life. We hope that by shedding light on the factors contributing to adversarial growth our society will be better prepared for the upcoming events in this pandemic and in future prolonged negative society-level experiences.

## Data Availability Statement

The datasets for this study can be found on the Open Science Framework [https://osf.io/cx4yg/].

## Ethics Statement

The studies involving human participants were reviewed and approved by the local ethics board at UiT (2017/2019), by the Norwegian Centre for research data (ref nr.: 287376) covering Europe. The study was also approved by the local ethics committee at Ben Gurion University and by the ethics committee from Facultad de Ciencias Humanas Universidad Nacional de Colombia. Sede Bogotá (Ethical approval No. B.VIE-FCH-21-2020). The patients/participants provided their written informed consent to participate in this study.

## Author Contributions

GP and MM conceived and designed the study. KK, ND, NR, RD, and RT recruited participants and translated the material. GP performed the statistical analysis. GP, MM, and NR wrote the first draft of the manuscript. All authors contributed to manuscript revisions and read and approved the submitted version.

## Conflict of Interest

The authors declare that the research was conducted in the absence of any commercial or financial relationships that could be construed as a potential conflict of interest.

## Footnotes

^1^LRT, Loglikelihood ratio.
