# Supplementary material for: Identifying Resilience Factors of Distress and Paranoia During the COVID-19 Outbreak in Five Countries
Source: Front Psychol. 2021 Jun 10;12:661149. doi: 10.3389/fpsyg.2021.661149 (PMC8222673; doi:10.3389/fpsyg.2021.661149)
Supplement: Supplementary file 1 [file Data_Sheet_1.docx]

Supplementary Material for Mækelæ, Reggev et al. 2021: Identifying resilience factors of distress and paranoia during the COVID-19 outbreak in five countries

Table S1: COVID-19 situation during 2nd wave (cases, deaths, median age, life expectancy, proportion of people older than 70, and others) of the five countries (OurWorldInData.org).

|  | Brazil | Colombia | Germany | Israel | Norway |
| --- | --- | --- | --- | --- | --- |
| **April, 14.-28.4.** |  |  |  |  |  |
| Absolute number of cases | 73,235 | 5,949 | 159,912 | 15,672 | 7,660 |
| Relative number of cases per million inhabitants (rounded) | 345 | 117 | 1909 | 1811 | 1413 |
| Absolute number of deaths | 5083 | 269 | 6314 | 213 | 206 |
| Relative number of deaths per million inhabitants | 23.9 | 5.3 | 75.4 | 24.6 | 38 |
| Reproduction number | 1.5 | 1.3 | 0.67 | 0.55 | 0.7 |
| Stringency index | 77.3 | 90.7 | 76.9 | 87 | 67.6 |
| **July, 9.7.-8.8.** |  |  |  |  |  |
| Absolute number of cases | 3,012,412 | 376,870 | 216,903 | 824,81 | 9,599 |
| Relative number of cases per million inhabitants | 14,172 | 7407 | 2589 | 9529 | 1771 |
| Absolute number of deaths | 100,477 | 12,540 | 9201 | 659 | 256 |
| Relative number of deaths per million inhabitants | 472.7 | 246.5 | 109.8 | 76.1 | 47.2 |
| Reproduction number | 1.0 | 1.1 | 1.22 | 0.97 | 1.47 |
| Stringency index | 72.7 | 87.0 | 56.9 | 43.5 | 40.7 |
| **General characteristics** |  |  |  |  |  |
| Population Density | 25 | 44 | 237 | 403 | 14 |
| Median Age | 33.5 | 32.2 | 46.6 | 30.6 | 39.7 |
| % aged 70 or older | 5.1 | 4.3 | 16 | 7.4 | 10.8 |
| Life Expectancy | 75.9 | 77.3 | 81.3 | 83.0 | 82.4 |

Table S2: Reliability score for each scale per country

| Reliability scores (McDonalds omega) | Brazil | Colombia | Germany | Israel | Norway |
| --- | --- | --- | --- | --- | --- |
| Paranoia | 0.82 | 0.89 | 0.7 | 0.86 | 0.83 |
| General Distress | 0.87 | 0.84 | 0.86 | 0.87 | 0.9 |
| Perceived Risk | 0.82 | 0.84 | 0.91 | 0.83 | 0.85 |
| Trust | 0.95 | 0.96 | 0.97 | 0.96 | 0.94 |
| Thriving | 0.73 | 0.83 | 0.92 | 0.81 | 0.84 |
| Regularity | 0.73 | 0.75 | 0.75 | 0.77 | 0.76 |
| Perceived Efficacy of actions | 0.38 | 0.54 | 0.81 | 0.47 | 0.61 |
| Negative Mindset | 0.82 | 0.82 | 0.69 | 0.85 | 0.82 |
| Conspiracy score | 0.88 | 0.78 | 0.86 | 0.82 | 0.73 |
|  |  |  |  |  |  |

## Bivariate Spearman rank correlations

##
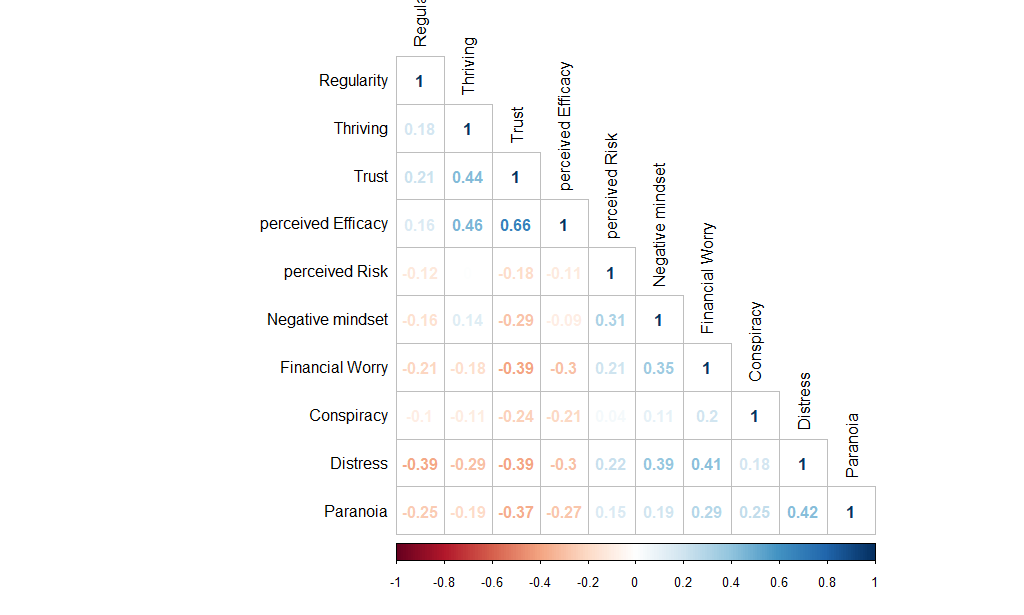


Figure S1: Spearman rank correlation among the 10 constructs

## Detailed statistics for Figure 1

| Table S3: Descriptive Statistics | | | | | |
| --- | --- | --- | --- | --- | --- |
|  | **Brazil (n=384)** | **Colombia (n=353)** | **Germany (n=273)** | **Israel (N=372)** | **Norway (n=832)** |
| Distress Mean | 12.052 | 13.986 | 10.897 | 10.33 | 7.654 |
| Distress Median | 11 | 13 | 9 | 9 | 6 |
| Distress SD | 6.588 | 7.076 | 7.265 | 6.158 | 6.187 |
| Paranoia Mean | 0.482 | 0.637 | 0.358 | 0.457 | 0.231 |
| Paranoia Median | 0.4 | 0.5 | 0.3 | 0.4 | 0.2 |
| Paranoia SD | 0.323 | 0.468 | 0.24 | 0.338 | 0.241 |
| Perceived Risk Mean | 29.56 | 24.777 | 17.898 | 21.619 | 20.876 |
| Perceived Risk Median | 28.6 | 23.4 | 14 | 21.1 | 19 |
| Perceived Risk SD | 15.637 | 14.218 | 16.775 | 12.901 | 13.102 |
| Regular Schedules Mean | 2.131 | 1.908 | 2.42 | 2.075 | 2.402 |
| Regular Schedules Median | 2 | 2 | 2.667 | 2 | 2.667 |
| Regular Schedules SD | 0.955 | 0.895 | 0.986 | 0.905 | 0.886 |
| Trust Mean | 1.268 | 1.256 | 2.197 | 2.067 | 3.252 |
| Trust Median | 1 | 1 | 2.25 | 2.125 | 3.5 |
| Trust SD | 0.805 | 0.833 | 1.176 | 0.875 | 0.706 |
| Thriving Mean | 3.097 | 3.021 | 2.453 | 3.152 | 3.357 |
| Thriving Median | 3.125 | 3 | 2.5 | 3.25 | 3.375 |
| Thriving SD | 0.499 | 0.541 | 0.876 | 0.537 | 0.489 |
| Conspiracy Mean | 17.201 | 29.496 | 24.286 | 17.637 | 8.345 |
| Conspiracy Median | 2.667 | 25 | 13.167 | 5.333 | 0.333 |
| Conspiracy SD | 25.653 | 25.231 | 27.93 | 24.835 | 16.038 |
| Negative Mindset Mean | 3.107 | 2.692 | 2.225 | 2.417 | 2.209 |
| Negative Mindset Median | 3.167 | 2.833 | 2.333 | 2.5 | 2.333 |
| Negative Mindset SD | 0.593 | 0.703 | 0.664 | 0.781 | 0.727 |
| Epistemic Belief Mean | 26.237 | 14.363 | 3.475 | 14.866 | 17.245 |
| Epistemic Belief Median | 20 | 10 | 1 | 10 | 11 |
| Epistemic Belief SD | 34.44 | 35.365 | 44.209 | 34.783 | 29.061 |
| Perceived Efficacy Mean | 2.206 | 2.49 | 2.342 | 2.854 | 3.284 |
| Perceived Efficacy Median | 2.333 | 2.667 | 2.667 | 3 | 3.333 |
| Perceived Efficacy SD | 0.768 | 0.76 | 1.006 | 0.656 | 0.572 |
| Financial Worry Mean | 62.197 | 59.259 | 44.822 | 45.777 | 27.204 |
| Financial Worry Median | 69 | 61 | 43 | 49 | 20 |
| Financial Worry SD | 29.961 | 27.422 | 33.352 | 31.254 | 27.033 |
| Outbreak lasting Mean | 3.851 | 4.129 | 4.1 | 3.457 | 4.455 |
| Outbreak lasting Median | 4 | 4 | 5 | 4 | 5 |
| Outbreak lasting SD | 0.955 | 0.835 | 1.538 | 1.238 | 0.766 |
| Restrictions lasting Mean | 2.623 | 2.96 | 3.349 | 2.403 | 3.404 |
| Restrictions lasting Median | 3 | 3 | 3 | 2 | 4 |
| Restrictions lasting SD | 1.067 | 1.054 | 1.297 | 1.181 | 1.006 |
|  |  |  |  |  |  |

Legend: for some scales the number of respondents is 1-7 lower than the N provided in the header

General distress was lowest in Norway (main effect of country: F(4,2937) = 81.93, p < .001, Tukey post-hoc: all comparisons with Norway p’s < .001, t’s > 5.65) and did not change over time (F(1,2937)=2.09, p = .149, η_p_^2^ = .001). Similarly, Norway had the lowest score of paranoia (F(4, 2934) = 113.1, p <. 001, η_p_^2^ = .134, Tukey post-hoc p’s < .001, all t’s > 4.93) and paranoia did not change over time (F(1, 2934) = 1.78, p = .182, η_p_^2^ = .001). Perceived efficacy was rated as high in Norway (Mean = 3.28, SD = .025) and low in Brazil (Mean = 2.21, SE = .036), yielding a large main effect of country (F(4, 2931) = 237.4, p < .001, η_p_^2^ = .245, Tukey post-hoc all p’s < .001, all t’s > 12.05) and for all countries but Germany perceived efficacy decreased from April to July (F(1, 2931) = 35.4, p < .001, η_p_^2^ = .012), resulting in a significant interaction effect (F(4, 2931) = 21.8, p <. 001, η_p_^2^ = .029). Thriving was high in most countries, though highest in Norway and lowest in Germany (F(1, 2923) = 79.95, p < .001, η_p_^2^ = .099) and decreased over time in all but Germany (interaction significant F(4, 2923) = 7.28, p < .001, η_p_^2^ = .01, but no significant main effect for wave F(1, 2923) = 2.59, p = .107, η_p_^2^ = .001). Regularity was lowest in Colombia and on average increased slightly from April to July (main effect country: F(4, 2927) = 17.419, p < .001, η_p_^2^ = .027, main effect wave: F(1, 2927) = 5.286, p = .013, η_p_^2^ = .002). Regarding discriminating facts from conspiracy theories Norwegians endorsed least conspiracy theories among all five countries (F(4, 2195) = 68.5, p < .001, η_p_^2^ = .111, Tukey post-hoc Norway compared to the other four countries, all p’s < .001, t’s > 3.95). As for trust in their authorities, participants from Colombia and Brazil had the lowest score (F(4, 2934) = 704.275, p < .001, η_p_^2^ = .49) but overall trust did not change from April to July (F1, 2934) = .153, p = .695, η_p_^2^ = 0), Interestingly, though an interaction effect emerged, whereby trust increased among Germans but decreased among Israeli (F(4, 2934) = 16.908, p < .001, η_p_^2^ = .023). Brazilians had the most negative mindset (F(4, 2931) = 111.2, p < .001, η_p_^2^ = .132) but on average there was a reduction in negative mindset from April to July (F(1,2931) = 28.22, p < .001, η_p_^2^ = .01). Brazilians rated the pandemic more as predictable than did Germans (F(4, 2895) = 21.6, p < .001, η_p_^2^ = .029) and this rating slightly increased from April to July (F(1, 2895) = 7.08, p = .008, η_p_^2^ = .002), strongest in Israel and Brazil, yielding a significant interaction effect (F4, 2895) = 3.51, p = .007, η_p_^2^ = .005). Perceived risk of COVID-19 was highest in Brazil and lowest in Germany (F(4, 2924) = 53.14, p < .001, η_p_^2^ = .068) and did on average not change over time (F(1, 2924) = 3.46, p = .063, η_p_^2^ = .001), however perceived risk decreased in Germany and Norway but increased in Colombia and Israel, yielding a significant interaction effect (F(4, 2924) = 8.69, p < .001, η_p_^2^ = .012). Financial worries reflect the countries’ economy with Brazilians and Colombians having the largest worries (F4, 2805) = 130.16, p < .001, η_p_^2^ = .0157), though on average the worries reduced from April to July (F(1, 2805) = 49.67, p < .001, η_p_^2^ = .017), strongest in Germany, yielding a significant interaction effect (F(4,2805) = 2.75, p = .027, η_p_^2^ = .004). Regarding how long they think the restrictions will last Israeli rated the duration as shortest and Germans as longest (F(4, 2930) = 66.22, p < .001, η_p_^2^ = .073) and across all countries participants expected longer lasting restrictions when asked again in July (F(1, 2930) = 264.51, p < .001, η_p_^2^ = .083).

More details are provided in the tables below.

| **ANOVA – General distress** | | | | | | | | | | | | | |
| --- | --- | --- | --- | --- | --- | --- | --- | --- | --- | --- | --- | --- | --- |
| **Cases** | | **Sum of Squares** | | **df** | | **Mean Square** | | **F** | | **p** | | **η²** | |
| wave |  | 87.199 |  | 1.000 |  | 87.199 |  | 2.089 |  | 0.149 |  | 0.001 |  |
| Country |  | 13681.145 |  | 4.000 |  | 3420.286 |  | 81.925 |  | < .001 |  | 0.100 |  |
| wave ✻ Country |  | 561.455 |  | 4.000 |  | 140.364 |  | 3.362 |  | 0.009 |  | 0.004 |  |
| Residual |  | 122616.666 |  | 2937.000 |  | 41.749 |  |  |  |  |  |  |  |
|  | | | | | | | | | | | | | |
| Note.  Type III Sum of Squares | | | | | | | | | | | | | |

### Post Hoc Tests

| **Post Hoc Comparisons - Country** | | | | | | | | | | | | | |
| --- | --- | --- | --- | --- | --- | --- | --- | --- | --- | --- | --- | --- | --- |
|  | |  | | **Mean Difference** | | **SE** | | **t** | | **Cohen's d** | | **p _tukey_** | |
| Brazil |  | Colombia |  | -0.748 |  | 0.516 |  | -1.449 |  | -0.110 |  | 0.596 |  |
|  |  | Germany |  | 2.454 |  | 0.601 |  | 4.086 |  | 0.357 |  | < .001 |  |
|  |  | Israel |  | 1.490 |  | 0.553 |  | 2.695 |  | 0.232 |  | 0.055 |  |
|  |  | Norway |  | 5.287 |  | 0.433 |  | 12.197 |  | 0.844 |  | < .001 |  |
| Colombia |  | Germany |  | 3.202 |  | 0.575 |  | 5.572 |  | 0.454 |  | < .001 |  |
|  |  | Israel |  | 2.238 |  | 0.524 |  | 4.267 |  | 0.339 |  | < .001 |  |
|  |  | Norway |  | 6.035 |  | 0.397 |  | 15.213 |  | 0.949 |  | < .001 |  |
| Germany |  | Israel |  | -0.964 |  | 0.608 |  | -1.587 |  | -0.145 |  | 0.506 |  |
|  |  | Norway |  | 2.833 |  | 0.502 |  | 5.649 |  | 0.446 |  | < .001 |  |
| Israel |  | Norway |  | 3.797 |  | 0.443 |  | 8.570 |  | 0.619 |  | < .001 |  |
|  | | | | | | | | | | | | | |
| *Note.*  Cohen's d does not correct for multiple comparisons. | | | | | | | | | | | | | |

## ANOVA_paranoia (CAPE)

| **ANOVA - CAPE** | | | | | | | | | | | | | |
| --- | --- | --- | --- | --- | --- | --- | --- | --- | --- | --- | --- | --- | --- |
| **Cases** | | **Sum of Squares** | | **df** | | **Mean Square** | | **F** | | **p** | | **η²** | |
| wave |  | 0.174 |  | 1.000 |  | 0.174 |  | 1.785 |  | 0.182 |  | 0.001 |  |
| Country |  | 44.227 |  | 4.000 |  | 11.057 |  | 113.098 |  | < .001 |  | 0.133 |  |
| wave ✻ Country |  | 0.432 |  | 4.000 |  | 0.108 |  | 1.104 |  | 0.353 |  | 0.001 |  |
| Residual |  | 286.836 |  | 2934.000 |  | 0.098 |  |  |  |  |  |  |  |
|  | | | | | | | | | | | | | |
| Note.  Type III Sum of Squares | | | | | | | | | | | | | |

### Post Hoc Tests

| **Post Hoc Comparisons - Country** | | | | | | | | | | | | | |
| --- | --- | --- | --- | --- | --- | --- | --- | --- | --- | --- | --- | --- | --- |
|  | |  | | **Mean Difference** | | **SE** | | **t** | | **Cohen's d** | | **p _tukey_** | |
| Brazil |  | Colombia |  | -0.119 |  | 0.025 |  | -4.777 |  | -0.300 |  | < .001 |  |
|  |  | Germany |  | 0.133 |  | 0.029 |  | 4.567 |  | 0.445 |  | < .001 |  |
|  |  | Israel |  | 0.050 |  | 0.027 |  | 1.843 |  | 0.150 |  | 0.349 |  |
|  |  | Norway |  | 0.252 |  | 0.021 |  | 12.033 |  | 0.942 |  | < .001 |  |
| Colombia |  | Germany |  | 0.252 |  | 0.028 |  | 9.067 |  | 0.659 |  | < .001 |  |
|  |  | Israel |  | 0.169 |  | 0.026 |  | 6.618 |  | 0.424 |  | < .001 |  |
|  |  | Norway |  | 0.372 |  | 0.019 |  | 19.369 |  | 1.182 |  | < .001 |  |
| Germany |  | Israel |  | -0.083 |  | 0.030 |  | -2.817 |  | -0.280 |  | 0.039 |  |
|  |  | Norway |  | 0.120 |  | 0.024 |  | 4.933 |  | 0.497 |  | < .001 |  |
| Israel |  | Norway |  | 0.203 |  | 0.022 |  | 9.385 |  | 0.761 |  | < .001 |  |
|  | | | | | | | | | | | | | |
| *Note.*  Cohen's d does not correct for multiple comparisons. | | | | | | | | | | | | | |

| **ANOVA – perceived Efficacy** | | | | | | | | | | | | | |
| --- | --- | --- | --- | --- | --- | --- | --- | --- | --- | --- | --- | --- | --- |
| **Cases** | | **Sum of Squares** | | **df** | | **Mean Square** | | **F** | | **p** | | **η²** | |
| wave |  | 17.767 |  | 1.000 |  | 17.767 |  | 35.405 |  | < .001 |  | 0.009 |  |
| Country |  | 476.460 |  | 4.000 |  | 119.115 |  | 237.373 |  | < .001 |  | 0.237 |  |
| wave ✻ Country |  | 43.762 |  | 4.000 |  | 10.940 |  | 21.802 |  | < .001 |  | 0.022 |  |
| Residual |  | 1470.794 |  | 2931.000 |  | 0.502 |  |  |  |  |  |  |  |
|  | | | | | | | | | | | | | |
| Note.  Type III Sum of Squares | | | | | | | | | | | | | |

### Post Hoc Tests

| **Post Hoc Comparisons - Country** | | | | | | | | | | | | | |
| --- | --- | --- | --- | --- | --- | --- | --- | --- | --- | --- | --- | --- | --- |
|  | |  | | **Mean Difference** | | **SE** | | **t** | | **Cohen's d** | | **p _tukey_** | |
| Brazil |  | Colombia |  | -0.272 |  | 0.057 |  | -4.781 |  | -0.354 |  | < .001 |  |
|  |  | Germany |  | -0.531 |  | 0.066 |  | -8.041 |  | -0.606 |  | < .001 |  |
|  |  | Israel |  | -0.458 |  | 0.061 |  | -7.521 |  | -0.616 |  | < .001 |  |
|  |  | Norway |  | -1.193 |  | 0.048 |  | -25.005 |  | -1.885 |  | < .001 |  |
| Colombia |  | Germany |  | -0.259 |  | 0.063 |  | -4.103 |  | -0.293 |  | < .001 |  |
|  |  | Israel |  | -0.186 |  | 0.058 |  | -3.227 |  | -0.248 |  | 0.011 |  |
|  |  | Norway |  | -0.922 |  | 0.044 |  | -21.133 |  | -1.445 |  | < .001 |  |
| Germany |  | Israel |  | 0.072 |  | 0.067 |  | 1.085 |  | 0.084 |  | 0.814 |  |
|  |  | Norway |  | -0.663 |  | 0.055 |  | -12.052 |  | -0.955 |  | < .001 |  |
| Israel |  | Norway |  | -0.735 |  | 0.049 |  | -15.070 |  | -1.184 |  | < .001 |  |
|  | | | | | | | | | | | | | |
| *Note.*  Cohen's d does not correct for multiple comparisons. | | | | | | | | | | | | | |

| **ANOVA - Thriving** | | | | | | | | | | | | | |
| --- | --- | --- | --- | --- | --- | --- | --- | --- | --- | --- | --- | --- | --- |
| **Cases** | | **Sum of Squares** | | **df** | | **Mean Square** | | **F** | | **p** | | **η²** | |
| wave |  | 1.948 |  | 1.000 |  | 1.948 |  | 6.284 |  | 0.012 |  | 0.002 |  |
| Country |  | 121.486 |  | 4.000 |  | 30.371 |  | 97.970 |  | < .001 |  | 0.117 |  |
| wave ✻ Country |  | 11.914 |  | 4.000 |  | 2.979 |  | 9.608 |  | < .001 |  | 0.011 |  |
| Residual |  | 906.151 |  | 2923.000 |  | 0.310 |  |  |  |  |  |  |  |
|  | | | | | | | | | | | | | |
| Note.  Type III Sum of Squares | | | | | | | | | | | | | |

### Post Hoc Tests

| **Post Hoc Comparisons - Country** | | | | | | | | | | | | | |
| --- | --- | --- | --- | --- | --- | --- | --- | --- | --- | --- | --- | --- | --- |
|  | |  | | **Mean Difference** | | **SE** | | **t** | | **Cohen's d** | | **p _tukey_** | |
| Brazil |  | Colombia |  | 0.018 |  | 0.045 |  | 0.403 |  | 0.034 |  | 0.994 |  |
|  |  | Germany |  | 0.347 |  | 0.052 |  | 6.696 |  | 0.508 |  | < .001 |  |
|  |  | Israel |  | -0.100 |  | 0.048 |  | -2.076 |  | -0.189 |  | 0.231 |  |
|  |  | Norway |  | -0.381 |  | 0.037 |  | -10.193 |  | -0.781 |  | < .001 |  |
| Colombia |  | Germany |  | 0.329 |  | 0.050 |  | 6.639 |  | 0.471 |  | < .001 |  |
|  |  | Israel |  | -0.118 |  | 0.046 |  | -2.580 |  | -0.216 |  | 0.074 |  |
|  |  | Norway |  | -0.399 |  | 0.034 |  | -11.671 |  | -0.800 |  | < .001 |  |
| Germany |  | Israel |  | -0.446 |  | 0.053 |  | -8.467 |  | -0.640 |  | < .001 |  |
|  |  | Norway |  | -0.728 |  | 0.043 |  | -16.834 |  | -1.249 |  | < .001 |  |
| Israel |  | Norway |  | -0.281 |  | 0.039 |  | -7.273 |  | -0.568 |  | < .001 |  |
|  | | | | | | | | | | | | | |
| *Note.*  Cohen's d does not correct for multiple comparisons. | | | | | | | | | | | | | |

| **ANOVA – Regular Schedules** | | | | | | | | | | | | | |
| --- | --- | --- | --- | --- | --- | --- | --- | --- | --- | --- | --- | --- | --- |
| **Cases** | | **Sum of Squares** | | **df** | | **Mean Square** | | **F** | | **p** | | **η²** | |
| wave |  | 5.286 |  | 1.000 |  | 5.286 |  | 6.187 |  | 0.013 |  | 0.002 |  |
| Country |  | 69.675 |  | 4.000 |  | 17.419 |  | 20.387 |  | < .001 |  | 0.027 |  |
| wave ✻ Country |  | 7.966 |  | 4.000 |  | 1.992 |  | 2.331 |  | 0.054 |  | 0.003 |  |
| Residual |  | 2500.796 |  | 2927.000 |  | 0.854 |  |  |  |  |  |  |  |
|  | | | | | | | | | | | | | |
| Note.  Type III Sum of Squares | | | | | | | | | | | | | |

| **Post Hoc Comparisons - Country** | | | | | | | | | | | | | |
| --- | --- | --- | --- | --- | --- | --- | --- | --- | --- | --- | --- | --- | --- |
|  | |  | | **Mean Difference** | | **SE** | | **t** | | **Cohen's d** | | **p _tukey_** | |
| Brazil |  | Colombia |  | 0.049 |  | 0.074 |  | 0.655 |  | 0.052 |  | 0.966 |  |
|  |  | Germany |  | -0.268 |  | 0.086 |  | -3.115 |  | -0.277 |  | 0.016 |  |
|  |  | Israel |  | -0.073 |  | 0.079 |  | -0.917 |  | -0.078 |  | 0.890 |  |
|  |  | Norway |  | -0.373 |  | 0.062 |  | -5.991 |  | -0.403 |  | < .001 |  |
| Colombia |  | Germany |  | -0.317 |  | 0.082 |  | -3.850 |  | -0.336 |  | 0.001 |  |
|  |  | Israel |  | -0.121 |  | 0.075 |  | -1.615 |  | -0.133 |  | 0.488 |  |
|  |  | Norway |  | -0.422 |  | 0.057 |  | -7.414 |  | -0.462 |  | < .001 |  |
| Germany |  | Israel |  | 0.196 |  | 0.087 |  | 2.250 |  | 0.209 |  | 0.162 |  |
|  |  | Norway |  | -0.105 |  | 0.072 |  | -1.463 |  | -0.113 |  | 0.587 |  |
| Israel |  | Norway |  | -0.301 |  | 0.063 |  | -4.740 |  | -0.330 |  | < .001 |  |
|  | | | | | | | | | | | | | |
| *Note.*  Cohen's d does not correct for multiple comparisons. | | | | | | | | | | | | | |

| **ANOVA - Fact-Fake / Conspiracy Score** | | | | | | | | | | | | | |
| --- | --- | --- | --- | --- | --- | --- | --- | --- | --- | --- | --- | --- | --- |
| **Cases** | | **Sum of Squares** | | **df** | | **Mean Square** | | **F** | | **p** | | **η²** | |
| Country |  | 282620.985 |  | 4.000 |  | 70655.246 |  | 68.523 |  | < .001 |  | 0.111 |  |
| Residual |  | 2.263e +6 |  | 2195.000 |  | 1031.113 |  |  |  |  |  |  |  |
|  | | | | | | | | | | | | | |
| Note.  Type III Sum of Squares | | | | | | | | | | | | | |

| **Post Hoc Comparisons - Country** | | | | | | | | | | | | | |
| --- | --- | --- | --- | --- | --- | --- | --- | --- | --- | --- | --- | --- | --- |
|  | |  | | **Mean Difference** | | **SE** | | **t** | | **Cohen's d** | | **p _tukey_** | |
| Brazil |  | Colombia |  | 18.168 |  | 2.374 |  | 7.652 |  | 0.507 |  | < .001 |  |
|  |  | Germany |  | 21.926 |  | 2.556 |  | 8.579 |  | 0.558 |  | < .001 |  |
|  |  | Israel |  | 7.019 |  | 2.345 |  | 2.992 |  | 0.204 |  | 0.023 |  |
|  |  | Norway |  | -7.840 |  | 1.985 |  | -3.949 |  | -0.274 |  | < .001 |  |
| Colombia |  | Germany |  | 3.758 |  | 2.602 |  | 1.444 |  | 0.098 |  | 0.599 |  |
|  |  | Israel |  | -11.149 |  | 2.396 |  | -4.654 |  | -0.336 |  | < .001 |  |
|  |  | Norway |  | -26.008 |  | 2.044 |  | -12.721 |  | -0.945 |  | < .001 |  |
| Germany |  | Israel |  | -14.907 |  | 2.576 |  | -5.787 |  | -0.407 |  | < .001 |  |
|  |  | Norway |  | -29.766 |  | 2.253 |  | -13.212 |  | -1.008 |  | < .001 |  |
| Israel |  | Norway |  | -14.859 |  | 2.011 |  | -7.389 |  | -0.561 |  | < .001 |  |
|  | | | | | | | | | | | | | |
| *Note.*  Cohen's d does not correct for multiple comparisons. | | | | | | | | | | | | | |

| **ANOVA – Trust in Authorities** | | | | | | | | | | | | | |
| --- | --- | --- | --- | --- | --- | --- | --- | --- | --- | --- | --- | --- | --- |
| **Cases** | | **Sum of Squares** | | **df** | | **Mean Square** | | **F** | | **p** | | **η²** | |
| wave |  | 0.101 |  | 1.000 |  | 0.101 |  | 0.153 |  | 0.695 |  | 0.000 |  |
| Country |  | 1858.293 |  | 4.000 |  | 464.573 |  | 704.275 |  | < .001 |  | 0.484 |  |
| wave ✻ Country |  | 44.612 |  | 4.000 |  | 11.153 |  | 16.908 |  | < .001 |  | 0.012 |  |
| Residual |  | 1935.406 |  | 2934.000 |  | 0.660 |  |  |  |  |  |  |  |
|  | | | | | | | | | | | | | |
| Note.  Type III Sum of Squares | | | | | | | | | | | | | |

| **Post Hoc Comparisons - Country** | | | | | | | | | | | | | |
| --- | --- | --- | --- | --- | --- | --- | --- | --- | --- | --- | --- | --- | --- |
|  | |  | | **Mean Difference** | | **SE** | | **t** | | **Cohen's d** | | **p _tukey_** | |
| Brazil |  | Colombia |  | -0.116 |  | 0.065 |  | -1.792 |  | -0.146 |  | 0.378 |  |
|  |  | Germany |  | -1.440 |  | 0.076 |  | -19.077 |  | -1.493 |  | < .001 |  |
|  |  | Israel |  | -0.732 |  | 0.070 |  | -10.502 |  | -0.878 |  | < .001 |  |
|  |  | Norway |  | -2.168 |  | 0.055 |  | -39.783 |  | -3.049 |  | < .001 |  |
| Colombia |  | Germany |  | -1.324 |  | 0.072 |  | -18.331 |  | -1.348 |  | < .001 |  |
|  |  | Israel |  | -0.615 |  | 0.066 |  | -9.308 |  | -0.723 |  | < .001 |  |
|  |  | Norway |  | -2.052 |  | 0.050 |  | -41.145 |  | -2.839 |  | < .001 |  |
| Germany |  | Israel |  | 0.709 |  | 0.077 |  | 9.257 |  | 0.694 |  | < .001 |  |
|  |  | Norway |  | -0.728 |  | 0.063 |  | -11.544 |  | -0.894 |  | < .001 |  |
| Israel |  | Norway |  | -1.436 |  | 0.056 |  | -25.678 |  | -1.933 |  | < .001 |  |
|  | | | | | | | | | | | | | |
| *Note.*  Cohen's d does not correct for multiple comparisons. | | | | | | | | | | | | | |

| **ANOVA – Negative Mindset** | | | | | | | | | | | | | |
| --- | --- | --- | --- | --- | --- | --- | --- | --- | --- | --- | --- | --- | --- |
| **Cases** | | **Sum of Squares** | | **df** | | **Mean Square** | | **F** | | **p** | | **η²** | |
| wave |  | 13.785 |  | 1.000 |  | 13.785 |  | 28.218 |  | < .001 |  | 0.008 |  |
| Country |  | 217.291 |  | 4.000 |  | 54.323 |  | 111.200 |  | < .001 |  | 0.130 |  |
| wave ✻ Country |  | 3.479 |  | 4.000 |  | 0.870 |  | 1.781 |  | 0.130 |  | 0.002 |  |
| Residual |  | 1431.832 |  | 2931.000 |  | 0.489 |  |  |  |  |  |  |  |
|  | | | | | | | | | | | | | |
| Note.  Type III Sum of Squares | | | | | | | | | | | | | |

| **Post Hoc Comparisons - Country** | | | | | | | | | | | | | |
| --- | --- | --- | --- | --- | --- | --- | --- | --- | --- | --- | --- | --- | --- |
|  | |  | | **Mean Difference** | | **SE** | | **t** | | **Cohen's d** | | **p _tukey_** | |
| Brazil |  | Colombia |  | 0.430 |  | 0.056 |  | 7.671 |  | 0.660 |  | < .001 |  |
|  |  | Germany |  | 0.932 |  | 0.065 |  | 14.323 |  | 1.490 |  | < .001 |  |
|  |  | Israel |  | 0.587 |  | 0.060 |  | 9.773 |  | 0.841 |  | < .001 |  |
|  |  | Norway |  | 0.902 |  | 0.047 |  | 19.150 |  | 1.314 |  | < .001 |  |
| Colombia |  | Germany |  | 0.502 |  | 0.062 |  | 8.071 |  | 0.730 |  | < .001 |  |
|  |  | Israel |  | 0.157 |  | 0.057 |  | 2.757 |  | 0.210 |  | 0.046 |  |
|  |  | Norway |  | 0.472 |  | 0.043 |  | 10.959 |  | 0.660 |  | < .001 |  |
| Germany |  | Israel |  | -0.345 |  | 0.066 |  | -5.241 |  | -0.465 |  | < .001 |  |
|  |  | Norway |  | -0.031 |  | 0.054 |  | -0.566 |  | -0.043 |  | 0.980 |  |
| Israel |  | Norway |  | 0.314 |  | 0.048 |  | 6.532 |  | 0.426 |  | < .001 |  |
|  | | | | | | | | | | | | | |
| *Note.*  Cohen's d does not correct for multiple comparisons. | | | | | | | | | | | | | |

| **ANOVA - Epistemic aleatory belief** | | | | | | | | | | | | | |
| --- | --- | --- | --- | --- | --- | --- | --- | --- | --- | --- | --- | --- | --- |
| **Cases** | | **Sum of Squares** | | **df** | | **Mean Square** | | **F** | | **p** | | **η²** | |
| wave |  | 7739.305 |  | 1.000 |  | 7739.305 |  | 7.085 |  | 0.008 |  | 0.002 |  |
| Country |  | 94388.435 |  | 4.000 |  | 23597.109 |  | 21.602 |  | < .001 |  | 0.029 |  |
| wave ✻ Country |  | 15319.626 |  | 4.000 |  | 3829.907 |  | 3.506 |  | 0.007 |  | 0.005 |  |
| Residual |  | 3.162e +6 |  | 2895.000 |  | 1092.368 |  |  |  |  |  |  |  |
|  | | | | | | | | | | | | | |
| Note.  Type III Sum of Squares | | | | | | | | | | | | | |

| **Post Hoc Comparisons - Country** | | | | | | | | | | | | | |
| --- | --- | --- | --- | --- | --- | --- | --- | --- | --- | --- | --- | --- | --- |
|  | |  | | **Mean Difference** | | **SE** | | **t** | | **Cohen's d** | | **p _tukey_** | |
| Brazil |  | Colombia |  | 16.546 |  | 2.653 |  | 6.238 |  | 0.477 |  | < .001 |  |
|  |  | Germany |  | 26.998 |  | 3.079 |  | 8.769 |  | 0.714 |  | < .001 |  |
|  |  | Israel |  | 11.402 |  | 2.839 |  | 4.016 |  | 0.333 |  | < .001 |  |
|  |  | Norway |  | 15.539 |  | 2.223 |  | 6.991 |  | 0.522 |  | < .001 |  |
| Colombia |  | Germany |  | 10.452 |  | 2.952 |  | 3.541 |  | 0.271 |  | 0.004 |  |
|  |  | Israel |  | -5.145 |  | 2.701 |  | -1.905 |  | -0.147 |  | 0.315 |  |
|  |  | Norway |  | -1.008 |  | 2.043 |  | -0.493 |  | -0.033 |  | 0.988 |  |
| Germany |  | Israel |  | -15.597 |  | 3.121 |  | -4.998 |  | -0.409 |  | < .001 |  |
|  |  | Norway |  | -11.460 |  | 2.572 |  | -4.455 |  | -0.362 |  | < .001 |  |
| Israel |  | Norway |  | 4.137 |  | 2.280 |  | 1.814 |  | 0.138 |  | 0.365 |  |
|  | | | | | | | | | | | | | |
| *Note.*  Cohen's d does not correct for multiple comparisons. | | | | | | | | | | | | | |

| **ANOVA – perceived Risk (COVID-19)** | | | | | | | | | | | | | |
| --- | --- | --- | --- | --- | --- | --- | --- | --- | --- | --- | --- | --- | --- |
| **Cases** | | **Sum of Squares** | | **df** | | **Mean Square** | | **F** | | **p** | | **η²** | |
| wave |  | 663.235 |  | 1.000 |  | 663.235 |  | 3.460 |  | 0.063 |  | 0.001 |  |
| Country |  | 40738.527 |  | 4.000 |  | 10184.632 |  | 53.136 |  | < .001 |  | 0.067 |  |
| wave ✻ Country |  | 6665.318 |  | 4.000 |  | 1666.330 |  | 8.694 |  | < .001 |  | 0.011 |  |
| Residual |  | 560446.562 |  | 2924.000 |  | 191.671 |  |  |  |  |  |  |  |
|  | | | | | | | | | | | | | |
| Note.  Type III Sum of Squares | | | | | | | | | | | | | |

| **Post Hoc Comparisons - Country** | | | | | | | | | | | | | |
| --- | --- | --- | --- | --- | --- | --- | --- | --- | --- | --- | --- | --- | --- |
|  | |  | | **Mean Difference** | | **SE** | | **t** | | **Cohen's d** | | **p _tukey_** | |
| Brazil |  | Colombia |  | 2.447 |  | 1.108 |  | 2.208 |  | 0.162 |  | 0.177 |  |
|  |  | Germany |  | 12.529 |  | 1.288 |  | 9.725 |  | 0.796 |  | < .001 |  |
|  |  | Israel |  | 5.803 |  | 1.185 |  | 4.897 |  | 0.405 |  | < .001 |  |
|  |  | Norway |  | 10.611 |  | 0.929 |  | 11.418 |  | 0.784 |  | < .001 |  |
| Colombia |  | Germany |  | 10.082 |  | 1.234 |  | 8.170 |  | 0.659 |  | < .001 |  |
|  |  | Israel |  | 3.356 |  | 1.126 |  | 2.982 |  | 0.241 |  | 0.024 |  |
|  |  | Norway |  | 8.164 |  | 0.852 |  | 9.577 |  | 0.614 |  | < .001 |  |
| Germany |  | Israel |  | -6.726 |  | 1.303 |  | -5.161 |  | -0.467 |  | < .001 |  |
|  |  | Norway |  | -1.919 |  | 1.076 |  | -1.783 |  | -0.142 |  | 0.384 |  |
| Israel |  | Norway |  | 4.808 |  | 0.950 |  | 5.061 |  | 0.376 |  | < .001 |  |
|  | | | | | | | | | | | | | |
| *Note.*  Cohen's d does not correct for multiple comparisons. | | | | | | | | | | | | | |

| **ANOVA – financial worry** | | | | | | | | | | | | | |
| --- | --- | --- | --- | --- | --- | --- | --- | --- | --- | --- | --- | --- | --- |
| **Cases** | | **Sum of Squares** | | **df** | | **Mean Square** | | **F** | | **p** | | **η²** | |
| wave |  | 40438.676 |  | 1.000 |  | 40438.676 |  | 49.665 |  | < .001 |  | 0.015 |  |
| Country |  | 423907.007 |  | 4.000 |  | 105976.752 |  | 130.157 |  | < .001 |  | 0.154 |  |
| wave ✻ Country |  | 8964.063 |  | 4.000 |  | 2241.016 |  | 2.752 |  | 0.027 |  | 0.003 |  |
| Residual |  | 2.284e +6 |  | 2805.000 |  | 814.224 |  |  |  |  |  |  |  |
|  | | | | | | | | | | | | | |
| Note.  Type III Sum of Squares | | | | | | | | | | | | | |

| **Post Hoc Comparisons - Country** | | | | | | | | | | | | | |
| --- | --- | --- | --- | --- | --- | --- | --- | --- | --- | --- | --- | --- | --- |
|  | |  | | **Mean Difference** | | **SE** | | **t** | | **Cohen's d** | | **p _tukey_** | |
| Brazil |  | Colombia |  | 1.414 |  | 2.317 |  | 0.610 |  | 0.048 |  | 0.974 |  |
|  |  | Germany |  | 21.446 |  | 2.770 |  | 7.743 |  | 0.676 |  | < .001 |  |
|  |  | Israel |  | 11.242 |  | 2.503 |  | 4.492 |  | 0.366 |  | < .001 |  |
|  |  | Norway |  | 33.376 |  | 1.968 |  | 16.958 |  | 1.213 |  | < .001 |  |
| Colombia |  | Germany |  | 20.033 |  | 2.635 |  | 7.602 |  | 0.658 |  | < .001 |  |
|  |  | Israel |  | 9.828 |  | 2.353 |  | 4.177 |  | 0.332 |  | < .001 |  |
|  |  | Norway |  | 31.962 |  | 1.774 |  | 18.015 |  | 1.191 |  | < .001 |  |
| Germany |  | Israel |  | -10.204 |  | 2.800 |  | -3.645 |  | -0.322 |  | 0.003 |  |
|  |  | Norway |  | 11.930 |  | 2.335 |  | 5.110 |  | 0.430 |  | < .001 |  |
| Israel |  | Norway |  | 22.134 |  | 2.010 |  | 11.010 |  | 0.807 |  | < .001 |  |
|  | | | | | | | | | | | | | |
| *Note.*  Cohen's d does not correct for multiple comparisons. | | | | | | | | | | | | | |

| **ANOVA – expectation restriction lasting** | | | | | | | | | | | | | |
| --- | --- | --- | --- | --- | --- | --- | --- | --- | --- | --- | --- | --- | --- |
| **Cases** | | **Sum of Squares** | | **df** | | **Mean Square** | | **F** | | **p** | | **η²** | |
| wave |  | 303.008 |  | 1.000 |  | 303.008 |  | 264.514 |  | < .001 |  | 0.077 |  |
| Country |  | 264.896 |  | 4.000 |  | 66.224 |  | 57.811 |  | < .001 |  | 0.067 |  |
| wave ✻ Country |  | 10.439 |  | 4.000 |  | 2.610 |  | 2.278 |  | 0.059 |  | 0.003 |  |
| Residual |  | 3356.391 |  | 2930.000 |  | 1.146 |  |  |  |  |  |  |  |
|  | | | | | | | | | | | | | |
| Note.  Type III Sum of Squares | | | | | | | | | | | | | |

| **Post Hoc Comparisons - Country** | | | | | | | | | | | | | |
| --- | --- | --- | --- | --- | --- | --- | --- | --- | --- | --- | --- | --- | --- |
|  | |  | | **Mean Difference** | | **SE** | | **t** | | **Cohen's d** | | **p _tukey_** | |
| Brazil |  | Colombia |  | -0.332 |  | 0.086 |  | -3.863 |  | -0.301 |  | 0.001 |  |
|  |  | Germany |  | -0.873 |  | 0.100 |  | -8.743 |  | -0.733 |  | < .001 |  |
|  |  | Israel |  | 0.025 |  | 0.092 |  | 0.276 |  | 0.022 |  | 0.999 |  |
|  |  | Norway |  | -0.788 |  | 0.072 |  | -10.883 |  | -0.736 |  | < .001 |  |
| Colombia |  | Germany |  | -0.541 |  | 0.095 |  | -5.685 |  | -0.460 |  | < .001 |  |
|  |  | Israel |  | 0.358 |  | 0.087 |  | 4.094 |  | 0.306 |  | < .001 |  |
|  |  | Norway |  | -0.456 |  | 0.066 |  | -6.931 |  | -0.429 |  | < .001 |  |
| Germany |  | Israel |  | 0.899 |  | 0.101 |  | 8.891 |  | 0.711 |  | < .001 |  |
|  |  | Norway |  | 0.085 |  | 0.083 |  | 1.028 |  | 0.077 |  | 0.842 |  |
| Israel |  | Norway |  | -0.813 |  | 0.074 |  | -10.986 |  | -0.734 |  | < .001 |  |
|  | | | | | | | | | | | | | |
| *Note.*  Cohen's d does not correct for multiple comparisons. | | | | | | | | | | | | | |

## Section 3.1. and 3.2.: Supplementary analysis for Hypothesis 1 and 2

## Analysis of distress and paranoia across all respondents (not restricted to the five countries)

## Linear Regression for general distress, April, across all countries.

| **Model Summary** | | | | | | | | | |  |  |  |  |  |  |  |
| --- | --- | --- | --- | --- | --- | --- | --- | --- | --- | --- | --- | --- | --- | --- | --- | --- |
| **Model** | | **R** | | **R²** | | **Adjusted R²** | | **RMSE** | |  |  |  |  |  |  |  |
| 1 |  | 0.654 |  | 0.427 |  | 0.425 |  | 5.321 |  |  |  |  |  |  |  |  |
|  | | | | | | | | | |  |  |  |  |  |  |  |
| **ANOVA** | | | | | | | | | | | | | | | | |
|  | |  | | | **Sum of Squares** | | | | **df** | | **Mean Square** | | **F** | | **p** | |
|  |  | Regression | |  | 48887.071 | | |  | 10 |  | 4888.707 |  | 172.688 |  | < .001 |  |
|  |  | Residual | |  | 65564.601 | | |  | 2316 |  | 28.309 |  |  |  |  |  |
|  |  | Total | |  | 114451.672 | | |  | 2326 |  |  |  |  |  |  |  |
|  | | | | | | | | | | | | | | | | |

| **Coefficients** | | | | | | | | | | | | | | | | | |
| --- | --- | --- | --- | --- | --- | --- | --- | --- | --- | --- | --- | --- | --- | --- | --- | --- | --- |
|  | | | | | | | | | | | | | | **95% CI** | | | |
|  | |  | | **Unstandardized** | | **Standard Error** | | **Standardized** | | **t** | | **p** | | **Lower** | | **Upper** | |
|  |  | (Intercept) |  | 11.780 |  | 0.816 |  |  |  | 14.440 |  | < .001 |  | 10.180 |  | 13.379 |  |
|  |  | Regular Schedules |  | -1.643 |  | 0.126 |  | -0.219 |  | -13.092 |  | < .001 |  | -1.890 |  | -1.397 |  |
|  |  | Thriving |  | -2.229 |  | 0.218 |  | -0.200 |  | -10.242 |  | < .001 |  | -2.656 |  | -1.802 |  |
|  |  | Trust in Authorities |  | -0.126 |  | 0.135 |  | -0.021 |  | -0.934 |  | 0.350 |  | -0.392 |  | 0.139 |  |
|  |  | Worry_financial |  | 0.033 |  | 0.004 |  | 0.151 |  | 8.249 |  | < .001 |  | 0.025 |  | 0.041 |  |
|  |  | CAPE |  | 4.925 |  | 0.335 |  | 0.256 |  | 14.717 |  | < .001 |  | 4.269 |  | 5.582 |  |
|  |  | Negative Mindset |  | 1.996 |  | 0.173 |  | 0.219 |  | 11.556 |  | < .001 |  | 1.657 |  | 2.335 |  |
|  |  | Risk |  | 0.026 |  | 0.008 |  | 0.054 |  | 3.219 |  | 0.001 |  | 0.010 |  | 0.042 |  |
|  |  | Perceived Efficacy |  | -0.023 |  | 0.183 |  | -0.003 |  | -0.126 |  | 0.900 |  | -0.383 |  | 0.337 |  |
|  |  | Gender |  | 1.477 |  | 0.248 |  | 0.096 |  | 5.950 |  | < .001 |  | 0.990 |  | 1.964 |  |
|  |  | Education |  | -0.144 |  | 0.127 |  | -0.018 |  | -1.129 |  | 0.259 |  | -0.393 |  | 0.106 |  |
|  | | | | | | | | | | | | | | | | | |

## Linear Regression for general distress, July, across all countries

| **Model Summary** | | | | | | | | | | |  |  |  |  |  |  |
| --- | --- | --- | --- | --- | --- | --- | --- | --- | --- | --- | --- | --- | --- | --- | --- | --- |
| **Model** | | **R** | | **R²** | | **Adjusted R²** | | **RMSE** | | |  |  |  |  |  |  |
| 1 |  | 0.631 |  | 0.398 |  | 0.386 |  | 5.020 | |  |  |  |  |  |  |  |
|  | | | | | | | | | | |  |  |  |  |  |  |
| **ANOVA** | | | | | | | | | | | | | | | | |
|  | |  | | | **Sum of Squares** | | | | **df** | | **Mean Square** | | **F** | | **p** | |
|  |  | Regression | |  | 8110.910 | | |  | 10 |  | 811.091 |  | 32.180 |  | < .001 |  |
|  |  | Residual | |  | 12249.597 | | |  | 486 |  | 25.205 |  |  |  |  |  |
|  |  | Total | |  | 20360.507 | | |  | 496 |  |  |  |  |  |  |  |
|  | | | | | | | | | | | | | | | | |

| **Coefficients** | | | | | | | | | | | | | | | | | |
| --- | --- | --- | --- | --- | --- | --- | --- | --- | --- | --- | --- | --- | --- | --- | --- | --- | --- |
|  | | | | | | | | | | | | | | **95% CI** | | | |
|  | |  | | **Unstandardized** | | **Standard Error** | | **Standardized** | | **t** | | **p** | | **Lower** | | **Upper** | |
|  |  | (Intercept) |  | 14.208 |  | 1.902 |  |  |  | 7.470 |  | < .001 |  | 10.471 |  | 17.945 |  |
|  |  | Regular Schedules |  | -1.485 |  | 0.250 |  | -0.228 |  | -5.938 |  | < .001 |  | -1.977 |  | -0.994 |  |
|  |  | Thriving |  | -2.220 |  | 0.517 |  | -0.191 |  | -4.293 |  | < .001 |  | -3.236 |  | -1.204 |  |
|  |  | Trust in Authorities |  | -0.811 |  | 0.333 |  | -0.115 |  | -2.434 |  | 0.015 |  | -1.465 |  | -0.156 |  |
|  |  | Worry_financial |  | 0.031 |  | 0.010 |  | 0.117 |  | 3.033 |  | 0.003 |  | 0.011 |  | 0.050 |  |
|  |  | CAPE |  | 2.959 |  | 0.984 |  | 0.122 |  | 3.007 |  | 0.003 |  | 1.026 |  | 4.892 |  |
|  |  | Negative Mindset |  | 1.980 |  | 0.375 |  | 0.217 |  | 5.277 |  | < .001 |  | 1.243 |  | 2.717 |  |
|  |  | Risk |  | 0.052 |  | 0.022 |  | 0.098 |  | 2.408 |  | 0.016 |  | 0.010 |  | 0.095 |  |
|  |  | Perceived Efficacy |  | 0.655 |  | 0.414 |  | 0.076 |  | 1.583 |  | 0.114 |  | -0.158 |  | 1.469 |  |
|  |  | Gender |  | 1.602 |  | 0.496 |  | 0.118 |  | 3.233 |  | 0.001 |  | 0.629 |  | 2.576 |  |
|  |  | Education |  | -0.756 |  | 0.297 |  | -0.095 |  | -2.547 |  | 0.011 |  | -1.339 |  | -0.173 |  |
|  | | | | | | | | | | | | | | | | | |

## Linear Regression for paranoia, April, across all countries

| **Model Summary** | | | | | | | | | |  |  |  |  |  |  |  |
| --- | --- | --- | --- | --- | --- | --- | --- | --- | --- | --- | --- | --- | --- | --- | --- | --- |
| **Model** | | **R** | | **R²** | | **Adjusted R²** | | **RMSE** | |  |  |  |  |  |  |  |
| 1 |  | 0.513 |  | 0.263 |  | 0.259 |  | 0.313 |  |  |  |  |  |  |  |  |
|  | | | | | | | | | |  |  |  |  |  |  |  |
| **ANOVA** | | | | | | | | | | | | | | | | |
| **Model** | |  | | | **Sum of Squares** | | | | **df** | | **Mean Square** | | **F** | | **p** | |
| 1 |  | Regression | |  | 80.365 | | |  | 11 |  | 7.306 |  | 74.686 |  | < .001 |  |
|  |  | Residual | |  | 225.577 | | |  | 2306 |  | 0.098 |  |  |  |  |  |
|  |  | Total | |  | 305.941 | | |  | 2317 |  |  |  |  |  |  |  |
|  | | | | | | | | | | | | | | | | |

| **Coefficients** | | | | | | | | | | | | | | | | | |
| --- | --- | --- | --- | --- | --- | --- | --- | --- | --- | --- | --- | --- | --- | --- | --- | --- | --- |
|  | | | | | | | | | | | | | | **95% CI** | | | |
|  | |  | | **Unstandardized** | | **Standard Error** | | **Standardized** | | **t** | | **p** | | **Lower** | | **Upper** | |
|  |  | (Intercept) |  | 0.405 |  | 0.050 |  |  |  | 8.030 |  | < .001 |  | 0.306 |  | 0.504 |  |
|  |  | Regular Schedules |  | -0.030 |  | 0.008 |  | -0.077 |  | -3.899 |  | < .001 |  | -0.045 |  | -0.015 |  |
|  |  | Thriving |  | 0.051 |  | 0.013 |  | 0.088 |  | 3.891 |  | < .001 |  | 0.025 |  | 0.077 |  |
|  |  | Trust in Authorities |  | -0.058 |  | 0.008 |  | -0.191 |  | -7.390 |  | < .001 |  | -0.074 |  | -0.043 |  |
|  |  | Financial Worry |  | 4.384e -4 |  | 2.393e -4 |  | 0.039 |  | 1.832 |  | 0.067 |  | -3.085e -5 |  | 9.076e -4 |  |
|  |  | Negative Mindset |  | -0.018 |  | 0.010 |  | -0.038 |  | -1.717 |  | 0.086 |  | -0.039 |  | 0.003 |  |
|  |  | Risk |  | 6.088e -4 |  | 4.815e -4 |  | 0.024 |  | 1.264 |  | 0.206 |  | -3.354e -4 |  | 0.002 |  |
|  |  | Perceived Efficacy |  | -0.001 |  | 0.011 |  | -0.003 |  | -0.127 |  | 0.899 |  | -0.023 |  | 0.020 |  |
|  |  | Gender |  | -0.073 |  | 0.015 |  | -0.092 |  | -5.000 |  | < .001 |  | -0.102 |  | -0.045 |  |
|  |  | Education |  | -0.032 |  | 0.008 |  | -0.079 |  | -4.221 |  | < .001 |  | -0.047 |  | -0.017 |  |
|  |  | CORE9 |  | 0.017 |  | 0.001 |  | 0.333 |  | 14.724 |  | < .001 |  | 0.015 |  | 0.020 |  |
|  |  | Conspiracy score |  | 0.001 |  | 2.947e -4 |  | 0.094 |  | 4.963 |  | < .001 |  | 8.849e -4 |  | 0.002 |  |
|  | | | | | | | | | | | | | | | | | |

## Section 3.1 and 3.2. Longitudinal subset

The longitudinal data, which had 525 participants, replicates the overall findings based on comparing over 2200 participants from April with over 700 from July, and also the cross-sectional pattern from March to July.

Among the 525 participants that used the same codeword (voluntarily) in April and July, we found no change in general distress, only a difference by country; wave: F(1, 458) = .758, p = .384, η_p_^2^ = 0; country: F(4, 458) = 25.589, p < .001, η_p_^2^ = .183, wave*country: F(4, 458) = 1.863, p = .116, η_p_^2^ = .002. Overall, paranoia did also not increase, though it did decrease in Colombia whereas it did increase in Brazil and Germany; wave: F(1, 458) = .007, p = .931, η_p_^2^ = 0; country: F(4, 458) = 29.794, p <. 001, η_p_^2^ = .206, wave*country: F(4, 458) = 4.399, p = .002, η_p_^2^ = .004. Finally, there was a reduction in perceived risk among German and Norwegian participants but no change for Brazilians, Colombians and Israeli, wave: F(1, 456) = 17.624, p < .001, η_p_^2^ = .009, country: F(4, 456) = 5.916, p < .001, η_p_^2^ = .011, wave*country: F(4, 456) = 20.774, p < .001, η_p_^2^ = .154.

## Section 3.5. Details for comparison to March data

At the beginning of the pandemic we launched a survey investigating the perceived efficacy of COVID-19 restrictions and how those affect mental health. We here compare general distress, paranoia, conspiracy score and perceived risk, four measures that were identical (distress and paranoia) or had overlapping items (conspiracy score, perceived risk) in the three surveys.

Distress did increase from March to April, particularly in Germany, and increased from April to July in Brazil and Israel, whereas it decreased in Colombia, Germany and Norway. Therefore both main effects and the interaction were significant, wave: F(2, 4671) = 11.851, p < .001, η_p_^2^ = .004; country: F(4, 4671) = 156.571, p < .001, η_p_^2^ = .117), wave*country: F(8, 4671) = 3.848, p < .001, η_p_^2^ = .006. Paranoia, on the other hand, decreased in all countries from March to April, decreased further in Colombia and Israel for July whereas paranoia in April was alike in July in Brazil, Germany and Norway. The two main effects were significant, but not the interaction, wave: F(2, 4677) = 16.189, p < .001, η_p_^2^ = .006. Country: F(4, 4677) = 220.952, p < .001, η_p_^2^ = .158. Notably, we found a large reduction in perceived risk from March to April, and also July was far below perceived risk in March. Both main effects and the interaction were significant, the interaction was driven by an increase in perceived risk in Colombia and Israel but decrease in Germany and Norway; wave: F(2, 4656) = 359.994, p < .001, η_p_^2^ = .129; country: F(4, 4656) = 18.33, p < .001, η_p_^2^ = .013; wave*country: F(8, 4656) = 16.944, p < .001, η_p_^2^ = .024. Finally, we compared the conspiracy score in March with April. Here we found no change for Brazil, an increase in Colombia, Israel and Norway and a decrease in Germany. This yielded significant main effects and interaction; wave: F(1,3891) = 11.981, p < .001, η_p_^2^ = .003; country: F(4, 3891) = 60.039, p < .001, η_p_^2^ = .057; wave*country: F(4, 3891) = 14.683, p < .001, η_p_^2^ = .014.

**ANOVA_general distress (9 items from CORE-OM)**

| **ANOVA – General Distress (CORE9)** | | | | | | | | | | | | | |
| --- | --- | --- | --- | --- | --- | --- | --- | --- | --- | --- | --- | --- | --- |
| **Cases** | | **Sum of Squares** | | **df** | | **Mean Square** | | **F** | | **p** | | **η²** | |
| survey |  | 919.903 |  | 2.000 |  | 459.952 |  | 11.851 |  | < .001 |  | 0.004 |  |
| Country |  | 24305.954 |  | 4.000 |  | 6076.488 |  | 156.571 |  | < .001 |  | 0.117 |  |
| survey ✻ Country |  | 1194.804 |  | 8.000 |  | 149.350 |  | 3.848 |  | < .001 |  | 0.006 |  |
| Residual |  | 181280.896 |  | 4671.000 |  | 38.810 |  |  |  |  |  |  |  |
|  | | | | | | | | | | | | | |
| *Note.*  Type III Sum of Squares | | | | | | | | | | | | | |

| **Descriptives – General distress** | | | | | | | | | |
| --- | --- | --- | --- | --- | --- | --- | --- | --- | --- |
| **survey** | | **Country** | | **Mean** | | **SD** | | **N** | |
| T0March |  | Brazil |  | 11.221 |  | 7.046 |  | 204 |  |
|  |  | Colombia |  | 13.902 |  | 6.805 |  | 420 |  |
|  |  | Germany |  | 7.495 |  | 5.126 |  | 99 |  |
|  |  | Israel |  | 9.886 |  | 5.593 |  | 281 |  |
|  |  | Norway |  | 6.839 |  | 4.932 |  | 735 |  |
| T1April |  | Brazil |  | 12.073 |  | 6.591 |  | 384 |  |
|  |  | Colombia |  | 14.048 |  | 7.088 |  | 353 |  |
|  |  | Germany |  | 10.875 |  | 7.261 |  | 273 |  |
|  |  | Israel |  | 10.361 |  | 6.180 |  | 371 |  |
|  |  | Norway |  | 7.680 |  | 6.243 |  | 832 |  |
| T2July |  | Brazil |  | 12.860 |  | 6.853 |  | 86 |  |
|  |  | Colombia |  | 12.381 |  | 6.448 |  | 118 |  |
|  |  | Germany |  | 9.150 |  | 6.709 |  | 60 |  |
|  |  | Israel |  | 11.593 |  | 6.160 |  | 81 |  |
|  |  | Norway |  | 6.679 |  | 5.764 |  | 389 |  |
|  | | | | | | | | | |


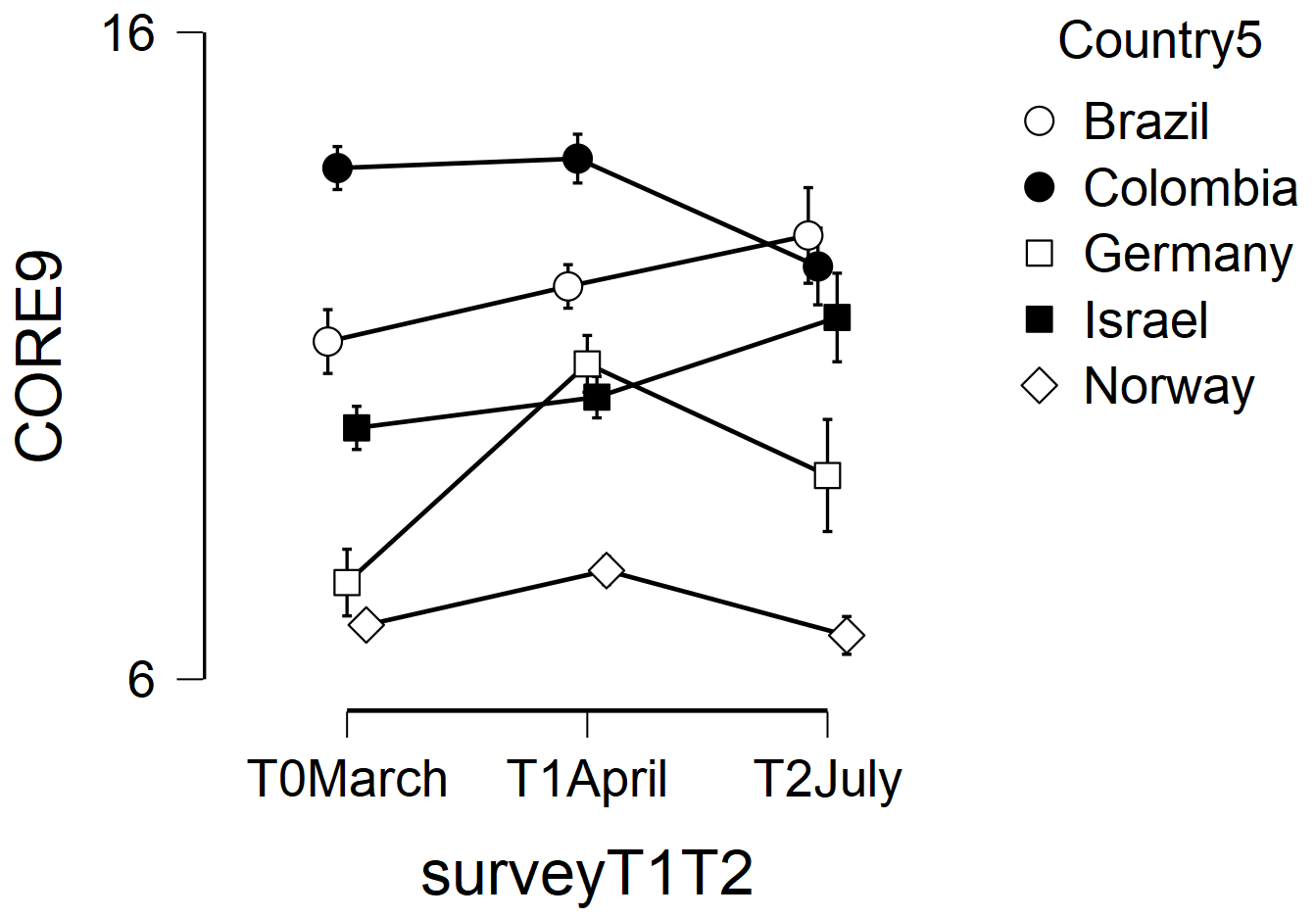


**ANOVA Paranoia (10 items from CAPE positive scale)**

| **ANOVA - Paranoia** | | | | | | | | | | | | | |
| --- | --- | --- | --- | --- | --- | --- | --- | --- | --- | --- | --- | --- | --- |
| **Cases** | | **Sum of Squares** | | **df** | | **Mean Square** | | **F** | | **p** | | **η²** | |
| survey |  | 3.022 |  | 2.000 |  | 1.511 |  | 16.189 |  | < .001 |  | 0.006 |  |
| Country |  | 82.488 |  | 4.000 |  | 20.622 |  | 220.952 |  | < .001 |  | 0.158 |  |
| survey✻ Country |  | 0.632 |  | 8.000 |  | 0.079 |  | 0.846 |  | 0.562 |  | 0.001 |  |
| Residual |  | 436.517 |  | 4677.000 |  | 0.093 |  |  |  |  |  |  |  |
|  | | | | | | | | | | | | | |
| *Note.*  Type III Sum of Squares | | | | | | | | | | | | | |

| **Descriptives - Paranoia** | | | | | | | | | |
| --- | --- | --- | --- | --- | --- | --- | --- | --- | --- |
| **survey** | | **Country** | | **Mean** | | **SD** | | **N** | |
| T0March |  | Brazil |  | 0.524 |  | 0.338 |  | 204 |  |
|  |  | Colombia |  | 0.698 |  | 0.368 |  | 421 |  |
|  |  | Germany |  | 0.434 |  | 0.265 |  | 99 |  |
|  |  | Israel |  | 0.514 |  | 0.309 |  | 289 |  |
|  |  | Norway |  | 0.282 |  | 0.219 |  | 735 |  |
| T1April |  | Brazil |  | 0.482 |  | 0.323 |  | 383 |  |
|  |  | Colombia |  | 0.639 |  | 0.467 |  | 353 |  |
|  |  | Germany |  | 0.356 |  | 0.240 |  | 273 |  |
|  |  | Israel |  | 0.459 |  | 0.339 |  | 371 |  |
|  |  | Norway |  | 0.231 |  | 0.242 |  | 832 |  |
| T2July |  | Brazil |  | 0.490 |  | 0.367 |  | 86 |  |
|  |  | Colombia |  | 0.572 |  | 0.417 |  | 118 |  |
|  |  | Germany |  | 0.350 |  | 0.270 |  | 60 |  |
|  |  | Israel |  | 0.414 |  | 0.280 |  | 79 |  |
|  |  | Norway |  | 0.236 |  | 0.235 |  | 389 |  |
|  | | | | | | | | | |


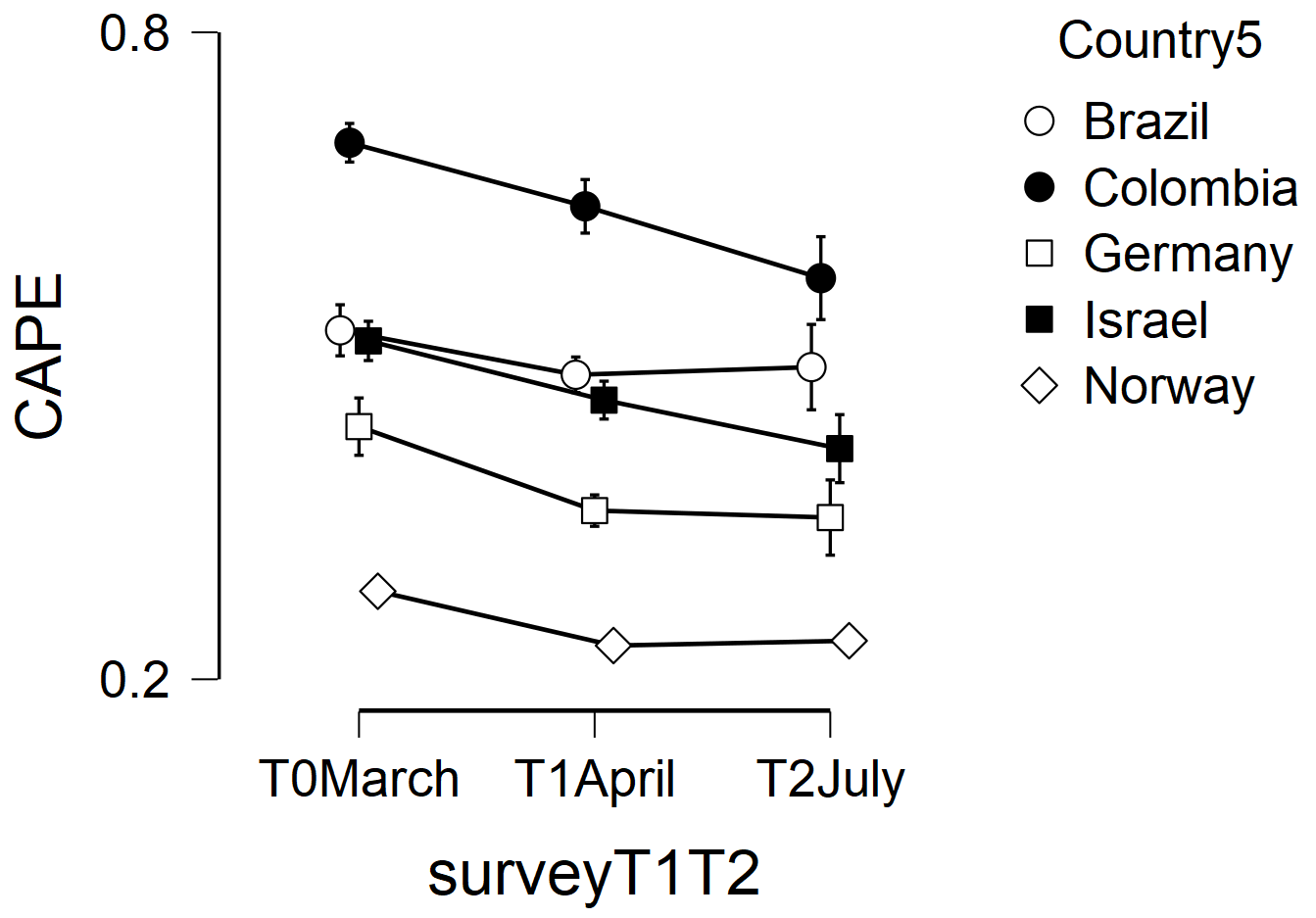


**ANOVA_perceived COVID-19 risk**

| **ANOVA - Risk_3items** | | | | | | | | | | | | | |
| --- | --- | --- | --- | --- | --- | --- | --- | --- | --- | --- | --- | --- | --- |
| **Cases** | | **Sum of Squares** | | **df** | | **Mean Square** | | **F** | | **p** | | **η²** | |
| survey |  | 207678.364 |  | 2.000 |  | 103839.182 |  | 359.994 |  | < .001 |  | 0.129 |  |
| Country |  | 21148.920 |  | 4.000 |  | 5287.230 |  | 18.330 |  | < .001 |  | 0.013 |  |
| survey ✻ Country |  | 39098.439 |  | 8.000 |  | 4887.305 |  | 16.944 |  | < .001 |  | 0.024 |  |
| Residual |  | 1.343e +6 |  | 4656.000 |  | 288.447 |  |  |  |  |  |  |  |
|  | | | | | | | | | | | | | |
| *Note.*  Type III Sum of Squares | | | | | | | | | | | | | |

| **Descriptives - Risk_3items** | | | | | | | | | |
| --- | --- | --- | --- | --- | --- | --- | --- | --- | --- |
| **survey** | | **Country** | | **Mean** | | **SD** | | **N** | |
| T0March |  | Brazil |  | 43.917 |  | 22.718 |  | 204 |  |
|  |  | Colombia |  | 33.546 |  | 20.824 |  | 416 |  |
|  |  | Germany |  | 40.141 |  | 19.324 |  | 99 |  |
|  |  | Israel |  | 39.332 |  | 20.136 |  | 287 |  |
|  |  | Norway |  | 41.924 |  | 20.512 |  | 734 |  |
| T1April |  | Brazil |  | 29.493 |  | 15.672 |  | 382 |  |
|  |  | Colombia |  | 24.912 |  | 14.404 |  | 352 |  |
|  |  | Germany |  | 18.818 |  | 17.280 |  | 266 |  |
|  |  | Israel |  | 21.700 |  | 12.977 |  | 369 |  |
|  |  | Norway |  | 23.977 |  | 14.527 |  | 829 |  |
| T2July |  | Brazil |  | 27.702 |  | 14.622 |  | 86 |  |
|  |  | Colombia |  | 27.417 |  | 15.627 |  | 117 |  |
|  |  | Germany |  | 14.230 |  | 12.545 |  | 60 |  |
|  |  | Israel |  | 23.889 |  | 13.104 |  | 81 |  |
|  |  | Norway |  | 15.080 |  | 10.836 |  | 389 |  |
|  | | | | | | | | | |


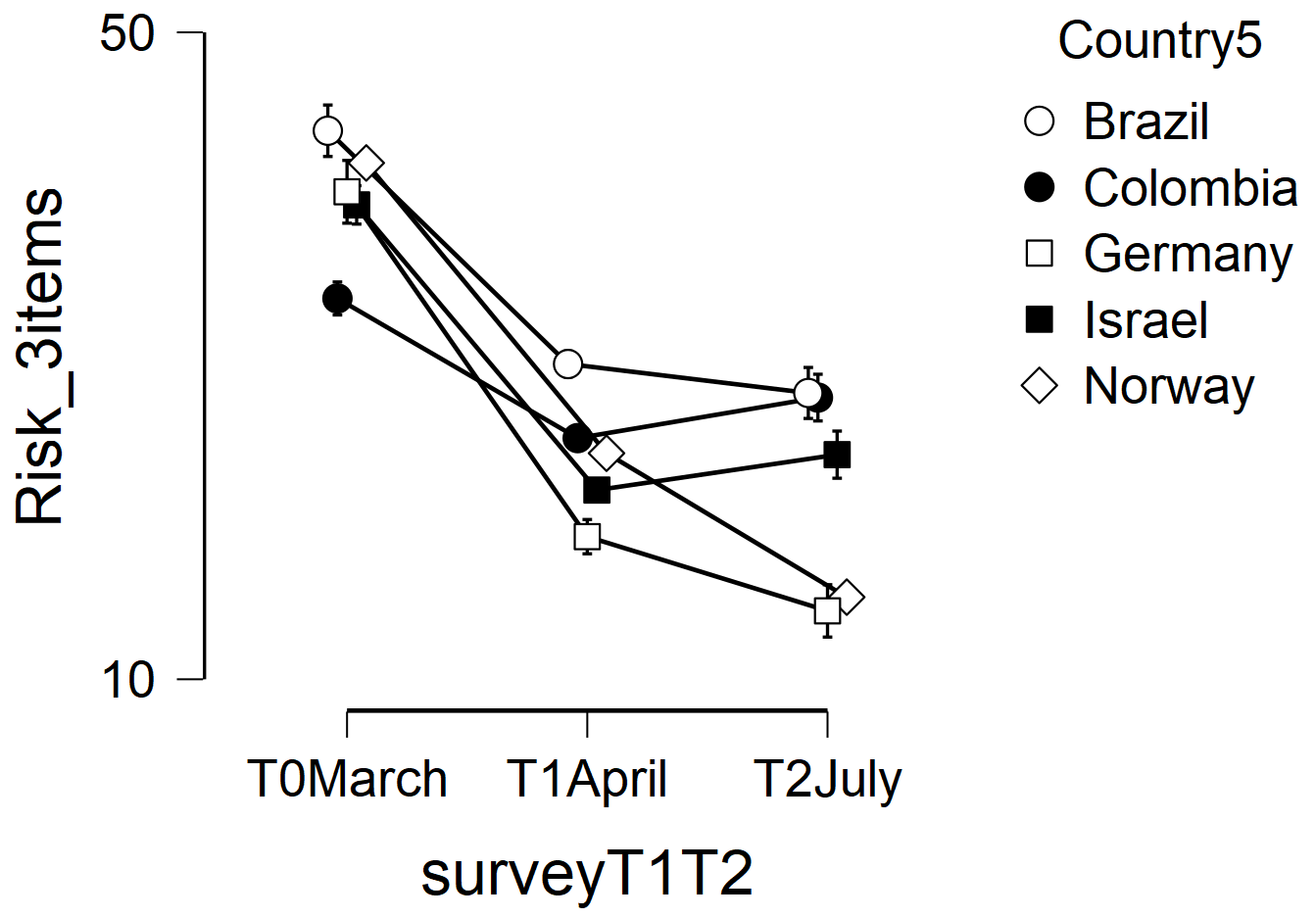


**ANOVA_Belief in conspiracy theories (vs factual knowledge)**

| **ANOVA - Fact-Fake** | | | | | | | | | | | | | |
| --- | --- | --- | --- | --- | --- | --- | --- | --- | --- | --- | --- | --- | --- |
| **Cases** | | **Sum of Squares** | | **df** | | **Mean Square** | | **F** | | **p** | | **η²** | |
| survey |  | 11625.126 |  | 1.000 |  | 11625.126 |  | 11.981 |  | < .001 |  | 0.003 |  |
| Country |  | 233017.038 |  | 4.000 |  | 58254.259 |  | 60.039 |  | < .001 |  | 0.057 |  |
| survey ✻ Country |  | 56986.715 |  | 4.000 |  | 14246.679 |  | 14.683 |  | < .001 |  | 0.014 |  |
| Residual |  | 3.775e +6 |  | 3891.000 |  | 970.279 |  |  |  |  |  |  |  |
|  | | | | | | | | | | | | | |
| *Note.*  Type III Sum of Squares | | | | | | | | | | | | | |

| **Descriptives - Fact-Fake** | | | | | | | | | |
| --- | --- | --- | --- | --- | --- | --- | --- | --- | --- |
| **survey** | | **Country** | | **Mean** | | **SD** | | **N** | |
| T0March |  | Brazil |  | 66.540 |  | 31.271 |  | 201 |  |
|  |  | Colombia |  | 37.580 |  | 33.420 |  | 420 |  |
|  |  | Germany |  | 55.469 |  | 28.397 |  | 98 |  |
|  |  | Israel |  | 52.603 |  | 31.320 |  | 278 |  |
|  |  | Norway |  | 54.911 |  | 29.397 |  | 705 |  |
| T1April |  | Brazil |  | 67.303 |  | 35.978 |  | 382 |  |
|  |  | Colombia |  | 50.851 |  | 32.216 |  | 351 |  |
|  |  | Germany |  | 43.228 |  | 41.351 |  | 269 |  |
|  |  | Israel |  | 57.198 |  | 29.574 |  | 367 |  |
|  |  | Norway |  | 68.893 |  | 25.013 |  | 830 |  |
|  | | | | | | | | | |


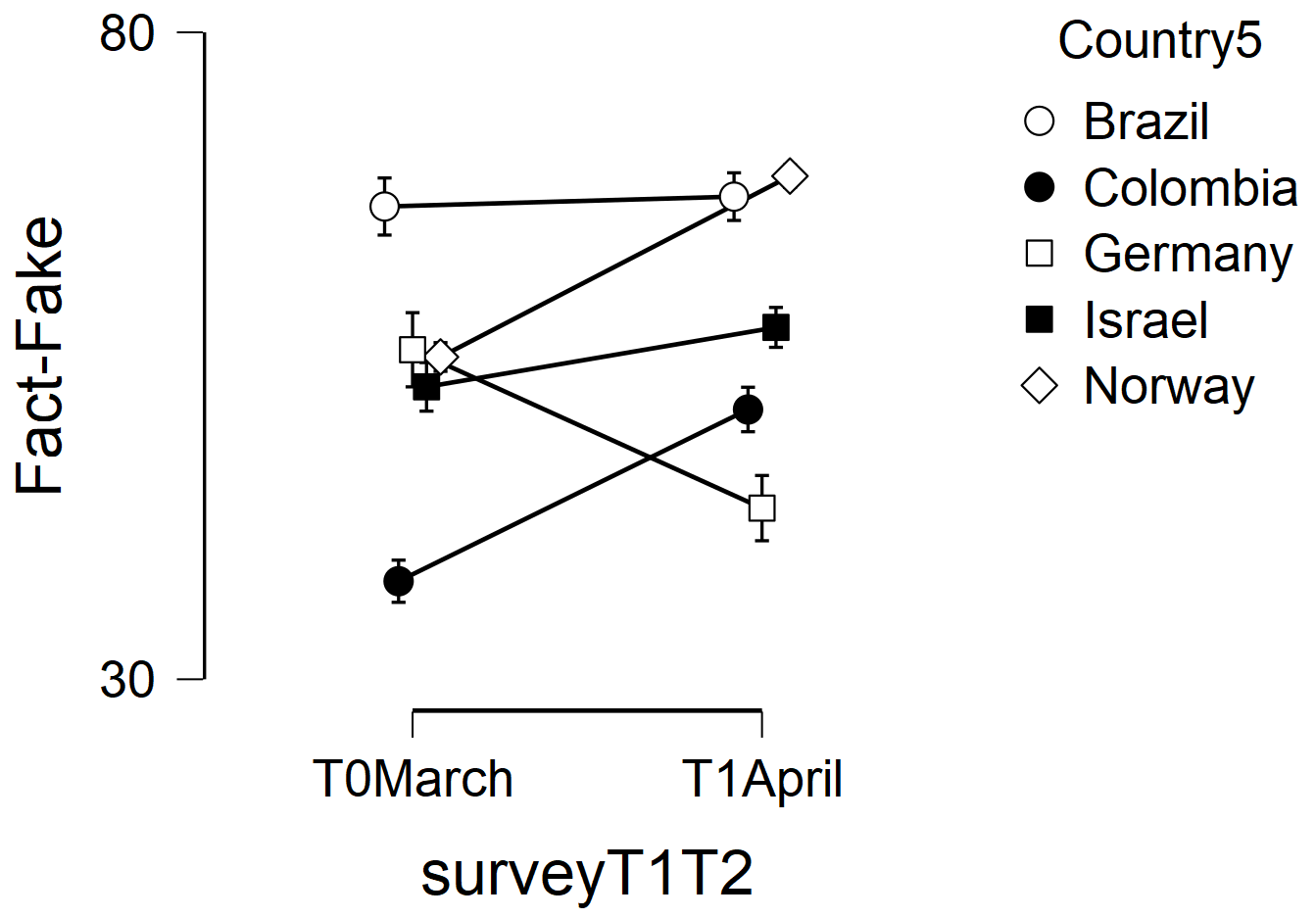


## ANOVA for perceived efficacy

| **ANOVA - perceivedEfficacy** | | | | | | | | | | | | | |
| --- | --- | --- | --- | --- | --- | --- | --- | --- | --- | --- | --- | --- | --- |
| **Cases** | | **Sum of Squares** | | **df** | | **Mean Square** | | **F** | | **p** | | **η²** | |
| survey |  | 27.082 |  | 2.000 |  | 13.541 |  | 26.918 |  | < .001 |  | 0.010 |  |
| Country |  | 535.411 |  | 4.000 |  | 133.853 |  | 266.090 |  | < .001 |  | 0.189 |  |
| survey ✻ Country |  | 78.899 |  | 8.000 |  | 9.862 |  | 19.606 |  | < .001 |  | 0.028 |  |
| Residual |  | 2186.697 |  | 4347.000 |  | 0.503 |  |  |  |  |  |  |  |
|  | | | | | | | | | | | | | |
| Note.  Type III Sum of Squares | | | | | | | | | | | | | |

| **Post Hoc Comparisons - Country** | | | | | | | | | | | | | | | | | |
| --- | --- | --- | --- | --- | --- | --- | --- | --- | --- | --- | --- | --- | --- | --- | --- | --- | --- |
|  | | | | | | **95% CI for Mean Difference** | | | |  | | | | | | | |
|  | |  | | **Mean Difference** | | **Lower** | | **Upper** | | **SE** | | **t** | | **Cohen's d** | | **p _tukey_** | |
| Brazil |  | Colombia |  | -0.220 |  | -0.338 |  | -0.103 |  | 0.043 |  | -5.108 |  | -0.283 |  | < .001 |  |
|  |  | Germany |  | -0.329 |  | -0.479 |  | -0.179 |  | 0.055 |  | -5.977 |  | -0.375 |  | < .001 |  |
|  |  | Israel |  | -0.445 |  | -0.572 |  | -0.319 |  | 0.046 |  | -9.624 |  | -0.586 |  | < .001 |  |
|  |  | Norway |  | -1.002 |  | -1.105 |  | -0.900 |  | 0.038 |  | -26.604 |  | -1.519 |  | < .001 |  |
| Colombia |  | Germany |  | -0.108 |  | -0.250 |  | 0.033 |  | 0.052 |  | -2.085 |  | -0.130 |  | 0.227 |  |
|  |  | Israel |  | -0.225 |  | -0.342 |  | -0.109 |  | 0.043 |  | -5.274 |  | -0.305 |  | < .001 |  |
|  |  | Norway |  | -0.782 |  | -0.873 |  | -0.692 |  | 0.033 |  | -23.566 |  | -1.195 |  | < .001 |  |
| Germany |  | Israel |  | -0.117 |  | -0.266 |  | 0.032 |  | 0.055 |  | -2.136 |  | -0.142 |  | 0.205 |  |
|  |  | Norway |  | -0.674 |  | -0.804 |  | -0.544 |  | 0.048 |  | -14.151 |  | -0.981 |  | < .001 |  |
| Israel |  | Norway |  | -0.557 |  | -0.659 |  | -0.455 |  | 0.037 |  | -14.959 |  | -0.880 |  | < .001 |  |
|  | | | | | | | | | | | | | | | | | |
| *Note.*  Cohen's d does not correct for multiple comparisons. | | | | | | | | | | | | | | | | | |
| *Note.*  Confidence level used: 0.95 | | | | | | | | | | | | | | | | | |

| **Descriptives - perceivedEfficacy** | | | | | | | | | |
| --- | --- | --- | --- | --- | --- | --- | --- | --- | --- |
| **survey** | | **Country** | | **Mean** | | **SD** | | **N** | |
| T0March |  | Brazil |  | 2.496 |  | 0.852 |  | 197 |  |
|  |  | Colombia |  | 2.613 |  | 0.721 |  | 419 |  |
|  |  | Germany |  | 2.420 |  | 0.843 |  | 70 |  |
|  |  | Israel |  | 2.916 |  | 0.683 |  | 273 |  |
|  |  | Norway |  | 3.116 |  | 0.626 |  | 462 |  |
| T1April |  | Brazil |  | 2.207 |  | 0.767 |  | 383 |  |
|  |  | Colombia |  | 2.488 |  | 0.765 |  | 353 |  |
|  |  | Germany |  | 2.348 |  | 1.009 |  | 273 |  |
|  |  | Israel |  | 2.854 |  | 0.655 |  | 371 |  |
|  |  | Norway |  | 3.283 |  | 0.573 |  | 831 |  |
| T2July |  | Brazil |  | 1.869 |  | 0.665 |  | 85 |  |
|  |  | Colombia |  | 2.131 |  | 0.741 |  | 117 |  |
|  |  | Germany |  | 2.789 |  | 0.968 |  | 60 |  |
|  |  | Israel |  | 2.137 |  | 0.757 |  | 80 |  |
|  |  | Norway |  | 3.180 |  | 0.580 |  | 388 |  |
|  | | | | | | | | | |


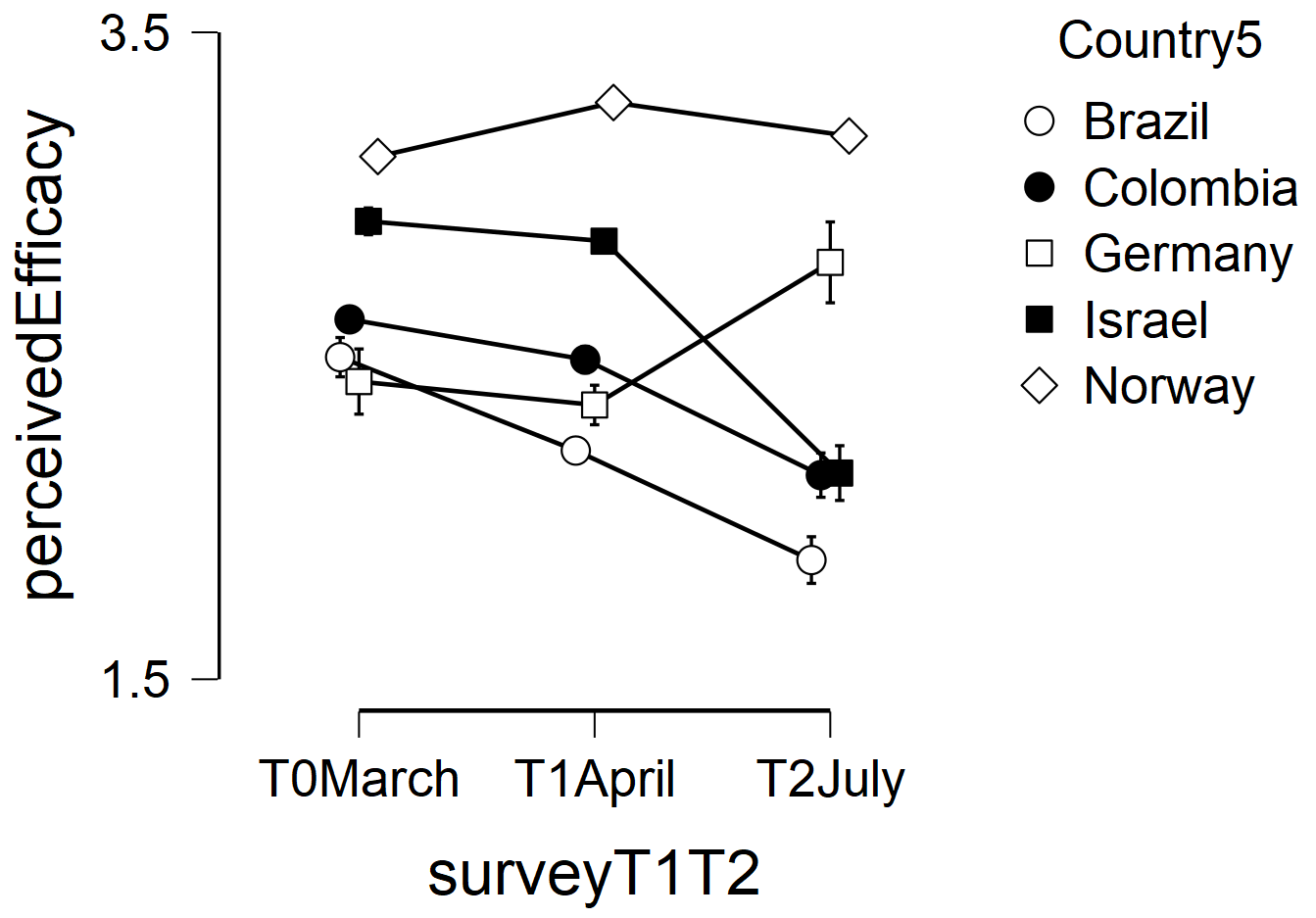


## ANOVA_negative mindset

| **ANOVA - NegativeFeelings** | | | | | | | | | | | | | |
| --- | --- | --- | --- | --- | --- | --- | --- | --- | --- | --- | --- | --- | --- |
| **Cases** | | **Sum of Squares** | | **df** | | **Mean Square** | | **F** | | **p** | | **η²** | |
| survey |  | 14.950 |  | 2.000 |  | 7.475 |  | 12.604 |  | < .001 |  | 0.005 |  |
| Country |  | 221.707 |  | 4.000 |  | 55.427 |  | 93.460 |  | < .001 |  | 0.079 |  |
| survey ✻ Country |  | 18.632 |  | 8.000 |  | 2.329 |  | 3.927 |  | < .001 |  | 0.007 |  |
| Residual |  | 2566.147 |  | 4327.000 |  | 0.593 |  |  |  |  |  |  |  |
|  | | | | | | | | | | | | | |
| Note.  Type III Sum of Squares | | | | | | | | | | | | | |

### Post Hoc Tests

| **Post Hoc Comparisons - Country** | | | | | | | | | | | | | | | | | |
| --- | --- | --- | --- | --- | --- | --- | --- | --- | --- | --- | --- | --- | --- | --- | --- | --- | --- |
|  | | | | | | **95% CI for Mean Difference** | | | |  | | | | | | | |
|  | |  | | **Mean Difference** | | **Lower** | | **Upper** | | **SE** | | **t** | | **Cohen's d** | | **p _tukey_** | |
| Brazil |  | Colombia |  | 0.434 |  | 0.306 |  | 0.561 |  | 0.047 |  | 9.279 |  | 0.607 |  | < .001 |  |
|  |  | Germany |  | 0.950 |  | 0.787 |  | 1.114 |  | 0.060 |  | 15.867 |  | 1.278 |  | < .001 |  |
|  |  | Israel |  | 0.405 |  | 0.268 |  | 0.543 |  | 0.050 |  | 8.059 |  | 0.532 |  | < .001 |  |
|  |  | Norway |  | 0.678 |  | 0.567 |  | 0.790 |  | 0.041 |  | 16.580 |  | 0.910 |  | < .001 |  |
| Colombia |  | Germany |  | 0.517 |  | 0.362 |  | 0.671 |  | 0.057 |  | 9.111 |  | 0.655 |  | < .001 |  |
|  |  | Israel |  | -0.028 |  | -0.155 |  | 0.098 |  | 0.046 |  | -0.612 |  | -0.036 |  | 0.973 |  |
|  |  | Norway |  | 0.245 |  | 0.146 |  | 0.343 |  | 0.036 |  | 6.781 |  | 0.318 |  | < .001 |  |
| Germany |  | Israel |  | -0.545 |  | -0.708 |  | -0.382 |  | 0.060 |  | -9.135 |  | -0.640 |  | < .001 |  |
|  |  | Norway |  | -0.272 |  | -0.414 |  | -0.130 |  | 0.052 |  | -5.230 |  | -0.342 |  | < .001 |  |
| Israel |  | Norway |  | 0.273 |  | 0.162 |  | 0.384 |  | 0.041 |  | 6.730 |  | 0.342 |  | < .001 |  |
|  | | | | | | | | | | | | | | | | | |
| *Note.*  Cohen's d does not correct for multiple comparisons. | | | | | | | | | | | | | | | | | |
| *Note.*  Confidence level used: 0.95 | | | | | | | | | | | | | | | | | |

| **Descriptives - NegativeFeelings** | | | | | | | | | |
| --- | --- | --- | --- | --- | --- | --- | --- | --- | --- |
| **survey** | | **Country** | | **Mean** | | **SD** | | **N** | |
| T0March |  | Brazil |  | 2.869 |  | 0.653 |  | 199 |  |
|  |  | Colombia |  | 2.489 |  | 0.741 |  | 421 |  |
|  |  | Germany |  | 1.760 |  | 0.829 |  | 68 |  |
|  |  | Israel |  | 2.409 |  | 0.830 |  | 264 |  |
|  |  | Norway |  | 2.024 |  | 0.762 |  | 451 |  |
| T1April |  | Brazil |  | 2.956 |  | 0.652 |  | 382 |  |
|  |  | Colombia |  | 2.518 |  | 0.759 |  | 353 |  |
|  |  | Germany |  | 1.963 |  | 0.883 |  | 273 |  |
|  |  | Israel |  | 2.496 |  | 0.818 |  | 371 |  |
|  |  | Norway |  | 2.343 |  | 0.783 |  | 830 |  |
| T2July |  | Brazil |  | 2.757 |  | 0.700 |  | 85 |  |
|  |  | Colombia |  | 2.274 |  | 0.751 |  | 117 |  |
|  |  | Germany |  | 2.008 |  | 0.795 |  | 60 |  |
|  |  | Israel |  | 2.462 |  | 1.003 |  | 80 |  |
|  |  | Norway |  | 2.180 |  | 0.732 |  | 388 |  |
|  | | | | | | | | | |


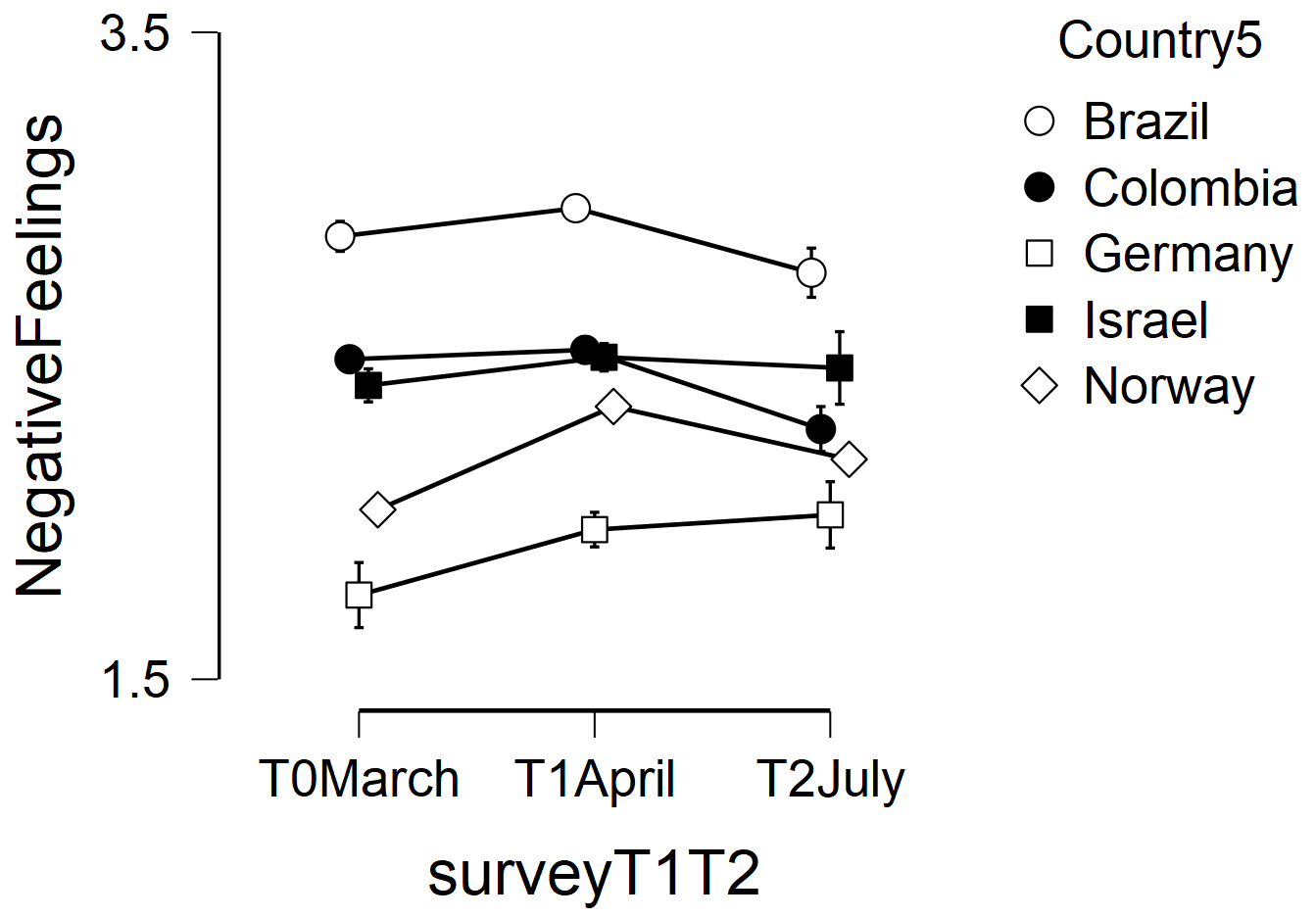


**Section 3.6.: Exploratory analysis of usual day activities on general distress**

In our pre-registration we included an average protective behaviour score as predictor in the mixed models. All activities but procrastination were added up to provide a sum score of protective activities. However, some of the items, such as caring for children, working from home and watching news may not necessarily affect general distress. Furthermore, some items referred to potentially irrelevant conditions, as parts of our sample were not necessarily employed or had children. We therefore looked at the 17 activities separately, correlating the amount spent doing them per day with the general distress score and correcting for multiple testing (significant if p <.003).

April: Across all five countries we found that working at the office or outside the house was negatively associated with general distress (ρ = -.141, p < .001) whereas working from home was not (ρ = -.05, p = .014). Additional protective activities included being some time of the day out of the house (ρ = -.107, p < .001), exercising (ρ = -.131, p < .001), and do-it-yourself type of activities (ρ = -.098, p < .001). Doing chores around the house (ρ = .03, p = .143), caring for children (ρ = -.047, p = .02), playing (ρ = .034, p = .095), mindfulness activity (ρ = .025, p = .209), and watching the news (ρ = .012, p = .54) had no effect on distress. Interestingly, helping friends was not associated with distress (ρ = -.017, p = .388), but too much phoning (ρ = .065, p = .001), communicating with friends (ρ = .166, p < .001) and providing emotional support (ρ = .078, p < .001) increased distress. . Finally, watching many films (ρ = .1, p < .001), too much praying (ρ = .079, p < .001), and procrastination (ρ = .249, p <. 001) were detrimental for distress. Figure 5 shows that procrastination had a negative effect on well-being in all countries, whereas exercising had a positive effect in all countries but Israel. Communicating with friends and family was not beneficial for general distress for participants from Colombia and Norway. Working outside the house was highly significant for Norway but not so in the other four countries, potentially due to inability to do so in some of the other countries at the time our sample was collected. Regarding praying, it was beneficial for people from Colombia only. Note, that most effect sizes were small.


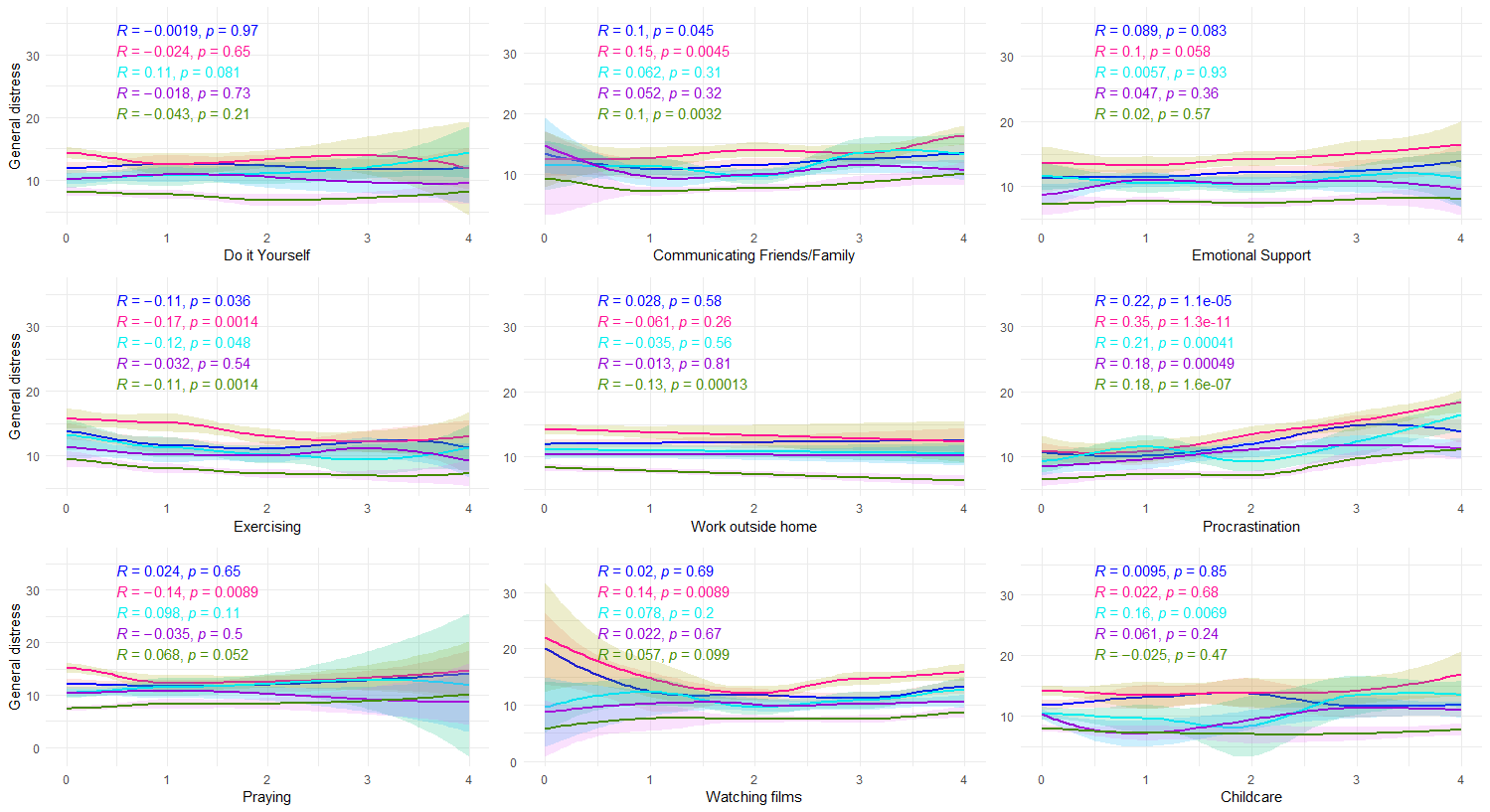
Figure S2: Plotting 9 of the 17 activities. Procrastinating, communicating with friends and family are not beneficial for mental health, whereas exercising is. X-axis is ordinal from 0 = not doing it at all, to 4 = doing it more than 5h a day. y-axis is general distress, color code: same as for Figure 2-4

July: Across all five countries we found that working at the office or outside the house was again negatively associated with general distress (ρ = -.22, p < .001) whereas working from home was positively associated (ρ = .153, p < .001). Helping friends was not associated with distress (ρ = -.037, p = .281), nor was phoning (ρ = .092, p = .008) or providing emotional support (ρ = .086, p < .013), but communicating with friends (ρ = .183, p < .001) increased distress. Being some time of the day out of the house (ρ = -.107, p = .002), and exercising (ρ = -.165, p < .001) was associated with less general distress. Doing the chores (ρ = -.019, p = .586) and caring for children (ρ = -.05, p = .143) had no effect on distress. Playing (ρ = -.021, p = .549) and watching the news (ρ = .048, p = .167) had also no effect on distress. Watching many films, (ρ = .129, p < .001) was increasing distress whereas DIY (ρ = -.103, p < .003) had no effect. Finally, mindfulness (ρ = .036, p = .294) and praying (ρ = .038, p < .276) had no effect on distress whereas procrastination (ρ = .257, p <. 001) was detrimental for distress. Looking at the significant relationships by country we found that working outside the home was beneficial in all but Israel. Communicating with friends and family was not associated with distress in Brazil and Colombia, but was so in Israel, Germany and Norway. Exercising was associated with less distress in all countries, strongest in Germany. Procrastination was also significant in all countries, strongest in Colombia.
